# ACSL4 Drives C5a/C5aR1–Calcium-Induced Fibroblast-to-Myofibroblast Transition in a Bleomycin-Induced Mouse Model of Pulmonary Fibrosis

**DOI:** 10.3390/biom15081106

**Published:** 2025-07-31

**Authors:** Tingting Ren, Jia Shi, Lili Zhuang, Ruiting Su, Yimei Lai, Niansheng Yang

**Affiliations:** Department of Rheumatology and Clinical Immunology, The First Affiliated Hospital of Sun Yat-Sen University, Guangzhou 510080, China

**Keywords:** idiopathic pulmonary fibrosis, ACSL4, fibroblast, myofibroblast, C5a/C5aR1, calcium signal

## Abstract

Idiopathic pulmonary fibrosis (IPF) is characterized by excessive extracellular matrix (ECM) deposition driven by aberrant fibroblast-to-myofibroblast transition (FMT). However, the upstream regulators and downstream effectors of this process remain incompletely understood. Here, we identify acyl-CoA synthetase long-chain family member 4 (ACSL4), a lipid metabolic enzyme, as a critical mediator linking complement component 5a (C5a)/C5a receptor 1 (C5aR1) signaling to FMT via calcium signaling. In bleomycin (BLM)-induced pulmonary fibrosis of C57BL/6JGpt mice, and in C5a-stimulated primary lung fibroblasts, the expression of ACSL4 was markedly upregulated. Pharmacological inhibition of ACSL4 (PRGL493) or C5aR1 (PMX53) attenuated the deposition of ECM and suppressed the expression of fibrotic markers in vivo and in vitro. Mechanistically, the activation of C5a/C5aR1 signaling increased intracellular calcium levels and promoted the expression of ACSL4, while inhibition of calcium signaling (FK506) reversed the upregulation of ACSL4 and FMT-related changes, including the expression of α-smooth muscle actin (αSMA) and the migration of fibroblasts. Notably, inhibition of ACSL4 did not affect the proliferation of fibroblasts, suggesting its specific role in phenotypic transition. These findings demonstrate that ACSL4 functions downstream of C5a/C5aR1-induced calcium signaling to promote FMT and the progression of pulmonary fibrosis. Targeting ACSL4 may therefore offer a novel therapeutic strategy for IPF.

## 1. Introduction

Idiopathic pulmonary fibrosis (IPF) is a progressive and ultimately fatal interstitial lung disease characterized by progressive dyspnea, restrictive lung function, and irreversible fibrotic remodeling [1]. Although anti-fibrotic therapies such as pirfenidone and nintedanib can slow the progression of IPF, they cannot completely control or reverse the process of fibrosis [2,3,4], highlighting the need for novel therapeutic strategies. Fibroblast-to-myofibroblast transition (FMT), a key process driving the deposition of extracellular matrix (ECM), is now recognized as a central event in the pathogenesis of IPF [5]. Recent studies have suggested that metabolic reprogramming, including altered lipid metabolism, may influence fibrotic response [6,7]. However, the specific metabolic enzymes that regulate FMT and contribute to fibrosis in IPF remain poorly defined.

Acyl-CoA synthetase long-chain family member 4 (ACSL4) is a key lipid metabolic enzyme that catalyzes the ligation of polyunsaturated fatty acids (PUFAs) with coenzyme A (CoA), thereby facilitating their entry into mitochondrial β-oxidation and lipid biosynthesis pathways [8,9,10,11]. Beyond its metabolic functions, ACSL4 has been implicated in various biological processes, including cell death, inflammation, and autophagy [12]. Recent studies suggest that ACSL4 may be involved in the occurrence of fibrotic diseases such as liver fibrosis [13,14,15], raising the possibility that it may play a role in pulmonary fibrogenesis. The potential role of ACSL4 in pulmonary fibrosis, particularly in regulating fibroblast activation and FMT, remains largely unexplored.

Complements are crucial immune system components that participate in innate and acquired immunity [16]. Complements can be activated via the classical, alternative, or lectin pathway, leading to the generation of complement component 5a (C5a), a potent anaphylatoxin that exerts strong pro-inflammatory and chemotactic effects [17]. The expression of C5a and C5a receptor 1 (C5aR1) increased in the lung tissues of IPF patients and mice with pulmonary fibrosis [18,19]. Although C5a/C5aR1 signaling has been implicated in pulmonary fibrosis [20], its precise role in regulating FMT within the lung microenvironment remains unclear.

C5a/C5aR1 signaling promotes the release of inflammatory mediators, including interleukin-1β (IL-1β), tumor necrosis factor α (TNFα), prostaglandins, and leukotrienes [21,22,23,24], several of which are derived from arachidonic acid metabolism. Notably, ACSL4, a lipid metabolic enzyme implicated in fibrotic diseases, preferentially activates arachidonic acid as a substrate. These observations raise the possibility that C5a/C5aR1 signaling may influence the activity of ACSL4 in the lungs and contribute to fibrotic progression. However, the relationship between C5a/C5aR1 signaling and ACSL4 in pulmonary fibrosis has not been elucidated.

In this study, we show that the expression of ACSL4 was elevated in lung tissues from bleomycin (BLM)-induced fibrosis models and positively correlated with α-smooth muscle actin (αSMA). Pharmacological inhibition of ACSL4 attenuated the differentiation of myofibroblasts and alleviated pulmonary fibrosis. Mechanistically, activation of C5a/C5aR1 signaling increased intracellular calcium levels, which, in turn, upregulated the expression of ACSL4. This calcium-dependent ACSL4 promoted FMT by enhancing the expression of αSMA and the migration of fibroblasts. Notably, blockade of calcium signaling with FK506 reversed both the upregulation of ACSL4 and the FMT-related changes, indicating that ACSL4 acts as a key downstream effector of C5a/C5aR1-mediated calcium signaling in fibrotic remodeling. These findings highlight a critical role for ACSL4 in FMT and the progression of fibrosis, suggesting that ACSL4 may serve as a promising therapeutic target in IPF.

## 2. Materials and Methods

### 2.1. Mice

Eight- to twelve-week-old male C57BL/6JGpt mice were purchased from GemPharmatech (Nanjing, China). The mice were specifically pathogen-free (SPF) and housed in the SPF Laboratory Animal Center of Sun Yat-sen University. The feeding room was kept at a 12 h light–12 h dark cycle, and the temperature was 22 ± 2 °C. The relevant operations were conducted in an SPF environment. The experimental protocol was approved by the Ethics Committee of Sun Yat-sen University (No. 2023002339), and all procedures were conducted following the guidelines of the National Institutes of Health Guide for Care and Use of Animals. The study was designed following the PREPARE guidelines [25] and reported in accordance with the ARRIVE 2.0 guidelines [26]. Animals were monitored daily for body weight and general well-being. Humane endpoints were based on sustained weight loss, clinical signs of respiratory distress, or moribund appearance.

### 2.2. Mouse Experiments

The BLM-induced mouse pulmonary fibrosis model was established as previously described [27,28,29]. Mice were anesthetized via intraperitoneal injection of 1% sodium pentobarbital (50 mg/kg), and the trachea was exposed through a neck incision. A 50 μL solution of BLM (MCE, New Jersey, USA, Cat#: HY-17565A, 3.2 U/kg), dissolved in PBS, was administered intratracheally. Control mice received an equal volume (50 μL) of PBS to serve as healthy controls for model validation. Immediately after injection, the mice were gently rotated to ensure uniform distribution of the solution throughout the lungs. Samples were collected on day 7 post-modeling to assess early fibrotic and inflammatory changes, and on day 21 to evaluate chronic fibrosis.

To study the role of ACSL4 in BLM-induced pulmonary fibrosis, the ACSL4 inhibitor PRGL493 (MCE, New Jersey, USA, Cat#: HY-13918, 250 μg/kg in 200 μL) or a vehicle (200 μL) was administered via intraperitoneal injection 1 h (h) before BLM exposure, and then once a day. To evaluate the role of C5a/C5aR1 signaling, the C5aR1 antagonist PMX53 (Selleck, Texas, USA, Cat#: S6239, 1 mg/kg in 200 μL) was used. For early-stage assessment, mice received a subcutaneous injection of PMX53 or saline 1 h before BLM instillation, and then once a day. For chronic-stage analysis, PMX53 or saline was given via subcutaneous injection once a day from day 7 after BLM instillation.

Mice were randomly assigned to experimental groups, with 5–8 animals per group, depending on the specific experiment. All drug administrations were performed in the morning hours (between 9:00 and 12:00 AM) to minimize circadian rhythm-related variability and ensure consistency across groups.

### 2.3. Extraction and Treatment of Lung Fibroblasts

Lung fibroblasts were isolated from 6- to 8-week-old C57BL/6JGpt mice. Mice were euthanized by cervical dislocation and immersed in 75% alcohol for 5 min. The trachea and blood vessels were carefully removed, and the lung tissues were minced into small fragments and placed in T25 culture flasks for culture. The medium was refreshed every two days. Cells reached 90% confluence at 10–12 days and then were passaged. Passage 1 (P1) lung fibroblasts were used for subsequent functional experiments. In some experiments, TGF-β1 (Sinobiological, Beijing, China, Cat#: 80116-RNAH-5), PRGL493, C5a (MCE, New Jersey, USA, Cat#: HY-P7695), or FK506 (Selleck, Texas, USA, Cat#: S5003) was added to the cell culture medium at the indicated concentrations and time points.

### 2.4. Real-Time Quantitative PCR (qPCR)

TRIzol was used to extract tissue-derived and cell-derived RNA. Reverse transcription was performed according to the instructions of the Evo-M-MLV RT Premix (Accurate Biology, Guangzhou, China, Cat#: AG11706), and qPCR was performed according to the instructions of SYBR Green Premix Pro Tag HS qPCR (Accurate Biology, Guangzhou, China, Cat#: AG11718). The relative RNA expression was normalized to that of β-actin, according to the 2^−ΔΔCT^ calculation method, and is shown as the mean ± SEM. The primer sequences are listed in Table S1.

### 2.5. Western Blot (WB)

Following homogenization with RIPA buffer, lung tissue and fibroblast samples underwent ice-based lysis and centrifugation, with subsequent supernatant collection for protein concentration quantification using a BCA assay kit (EpiZyme, Massachusetts, USA, Cat#: ZJ101). Protein electrophoresis was performed via 10% SDS-PAGE, and then the proteins were transferred to PVDF membranes (Merck Millipore, Darmstadt, Germany, Cat#: ISEQ00010). Following 1 h of blocking with 5% BSA at room temperature, the PVDF membranes were incubated with primary antibodies against ACSL4 (1:500; Affinity, Jiangsu, China, Cat#: DF12141), αSMA (1:1000; Abcam, Cambridge, UK, Cat#: ab5694), and β-actin (1:2000; Servicebio, Wuhan, China, Cat#: GB11001) at room temperature for 1 h, and then transferred to 4 °C overnight. The next day, a goat anti-rabbit secondary antibody (1:2000, Cell Signaling Technology, Massachusetts, USA, Cat#: 7074) containing HRP was incubated with the samples for 1 h at room temperature, and proteins were detected with Immobilon ECL Ultra Western HRP substrate (EpiZyme, Massachusetts, USA, Cat#: SQ201L). Images were analyzed via ImageJ (1.53t). Original figures can be found in Supplementary Materials. 

### 2.6. Pathological Staining of Lung Tissue

The left lung lobes of the mice were fixed in 4% paraformaldehyde for 24 h, followed by dehydration, paraffin embedding, and sectioning. Hematoxylin and eosin (H&E) staining and Masson staining were performed. Immunohistochemical staining was used to determine the expression of ACSL4, αSMA, and collagen I (COLI), and immunofluorescence was performed to assess the expression of CD3, vimentin, and CD88.

#### 2.6.1. H&E Staining

The sections were placed in hematoxylin for 3 min, differentiated with 1% hydrochloric alcohol for 3 s, and rinsed with running water for 1 min. The sections were placed in eosin for 5 min, dehydrated, and sealed with neutral gum. Eight areas were randomly selected from each section for the Ashcroft score, the Szapiel score, and nucleus counting. ImageJ was used to analyze the images.

#### 2.6.2. Immunohistochemical Staining

The sections were immersed in antigen repair solution and subjected to high heat for 30 min, and they were blocked with goat serum at room temperature for 1 h. The sections were incubated with antibodies against ACSL4 (1:200), αSMA (1:200), and COLI (1:200, Affinity, Jiangsu, China, Cat#: AF7001) for 1 h at room temperature and then transferred to 4 °C overnight. Then, they were incubated with endogenous peroxidase-blocking agents for 30 min, followed by a 1 h incubation with HRP-labeled anti-rabbit secondary antibody (1:500, Servicebio, Wuhan, China, Cat#: GB23303), and the expression of proteins was detected by DAB. The images were taken under a microscope and analyzed by ImageJ.

#### 2.6.3. Masson Staining

Staining was performed according to the instructions of the Masson staining kit (Servicebio, Wuhan, China, Cat#: G1006). In brief, the sections were immersed in solution A for 15 h and then incubated at 65 °C for 30 min. Then, they were stained with a B/C solution mixture for 1 min, followed by differentiation in 1% hydrochloric alcohol for 3 s, D solution staining for 6 min, and E solution differentiation for 1 min. Subsequently, they underwent F solution staining for 30 s, triple glacial acetic acid differentiation for 8 s each time, dehydration, and neutral gum sealing. The images were taken under a microscope and analyzed by ImageJ.

#### 2.6.4. Immunofluorescence Staining

After antigen repair, the sections were permeabilized with 0.5% Triton X-100 for 15 min, incubated with antibodies against CD3 (1:200, Abcam, Cambridge, UK, Cat#: Ab5690), vimentin (1:200, R&D, Minnesota, USA, Cat#: MAB2105-SP), and CD88 (1:200, Santa Cruz, Shanghai, China, Cat#: sc-53797, containing FITC labeling) for 1 h at room temperature, and then transferred to 4 °C overnight. The next day, AF488- and AF594-conjugated secondary antibodies (1:500, Thermo Fisher Scientific, Massachusetts, USA, Cat#: A-11008, A-11007) were used to label the anti-CD3 and anti-vimentin antibodies, respectively, for 30 min. Then, the sections were sealed with DAPI, and images were taken under a microscope.

### 2.7. Lung Coefficient

The lung coefficient was calculated by dividing lung weight (g) by body weight (kg), and it was used to evaluate pulmonary edema.

### 2.8. Magnetic Bead Particle Luminescence Method

An eight-item cytokine detection kit was used to detect cytokines in the peripheral blood. The reaction wells received 25 µL of solutions A/B/D/E (quality control/samples) and were incubated at 37 °C with shaking for 60 min, followed by three sequential magnetic separation cycles (1 min for each): first with 150 µL of LF solution, then 50 µL of C solution mixture after film-sealed shaking for 5 min at 37 °C, and finally 150 µL of F solution post-machine testing preparation for 30 s. Between each cycle, the supernatants were removed following magnetic separation. Dehydration steps were implemented using standardized magnetic bead clearance protocols.

### 2.9. Cell Immunofluorescence Staining

Fibroblasts were seeded in 96-well plates and fixed with methanol for 10 min. They were incubated with 0.1% Triton for 5 min, blocked with goat serum for 1 h, and labeled with an anti-vimentin antibody (1:200)/anti-ACSL4 antibody (1:200)/anti-αSMA antibody (1:200) for 1 h. On the following day, cells were sequentially labeled with AF488- or AF594-conjugated secondary antibodies (1:1000) targeting vimentin/ACSL4/αSMA under dark conditions for 30 min, followed by Hoechst nuclear counterstaining for 10 min. The images were taken under a fluorescence microscope.

### 2.10. Scratch Assay

The lung fibroblasts were harvested and cultured in DMEM containing 2% fetal bovine serum (FBS) for 8 h. A 10 µL tip was used to draw a line perpendicular to the transverse diameter of the plate, followed by washing with PBS. Cells were treated with either control medium, TGF-β1, TGF-β1 with PRGL493, C5a, C5a with PRGL493, or C5a with FK506. Wound closure was monitored at 0, 12, 24, and 48 h under a phase-contrast microscope and photographed. Using the ImageJ program, the size of the opened area was measured from the digital images. Four randomly selected images were acquired for each group.

### 2.11. Transwell Assay

The upper chamber mixture was serum-free DMEM with corresponding experimental interventions, and the lower chamber mixture was DMEM containing 20% FBS with corresponding interventions. Lung fibroblasts were added to the upper chambers and then placed in the cell incubator at 37 °C for 24 h. Following fixation with 4% paraformaldehyde for 10 min and permeabilization with 0.1% Triton X-100 for 10 min, transwell chambers were subjected to crystal violet staining for 30 min. The cells in the upper chamber were carefully removed. The images were taken under a microscope and analyzed by ImageJ.

### 2.12. Cell Viability and Proliferation

Lung fibroblasts were harvested and cultured in DMEM supplemented with 2% FBS for 8 h. Different concentrations of PRGL493 or FK506 were added to the wells, and the cells were cultured in a cell incubator at 37 °C for 24, 48, or 72 h. Then, 10 µL of CCK8 solution was added to each well, and the samples were cultured for another 3 h. The absorbance at 450 nm per well was measured via a multifunctional microplate reader. Cell viability was calculated according to the formula provided in the CCK8 instructions.

For cell proliferation, either control medium, TGF-β1, TGF-β1 with PRGL493, C5a, or C5a with PRGL493 was added to the wells for culture for 24, 48, and 72 h. Proliferation curves were constructed based on the OD values.

### 2.13. Calcium Imaging

To detect intracellular calcium flux, lung fibroblasts were labeled with Fluo-8AM (100 nM, AAT Bioquest, Pleasanton, CA, USA, Cat#: 21081) at 37 °C for 30 min and monitored by fluorescence microscope. The fluorescence intensity, exposure time, and gain were adjusted to start the video recording. After 30 s, ATP (100 nM, AAT Bioquest, Cat#: 21613), C5a (200 ng/mL), or HBSS was added to each well. Twenty-five cells were randomly selected for luminance profiling line analysis. The average value of each time point was plotted, and the highest value of each group was used for statistical analysis.

### 2.14. Statistical Methods

All data are presented as the mean ± SEM. Statistical analysis was performed using SPSS 25. Comparisons were assessed by unpaired Student’s *t*-test within two groups. To compare data between 3 or more groups, one-way or two-way ANOVA was conducted with or without repeated measurements, followed by a multiple comparisons post-test. Pearson correlation analysis was used according to the normal distribution of the quantitative data. Values of *p* < 0.05 were considered statistically significant in this study. The statistical methods are indicated in the figure legends for each panel.

## 3. Results

### 3.1. ACSL4 Expression Was Increased in Fibrotic Lung Tissues

**Figure 1:**
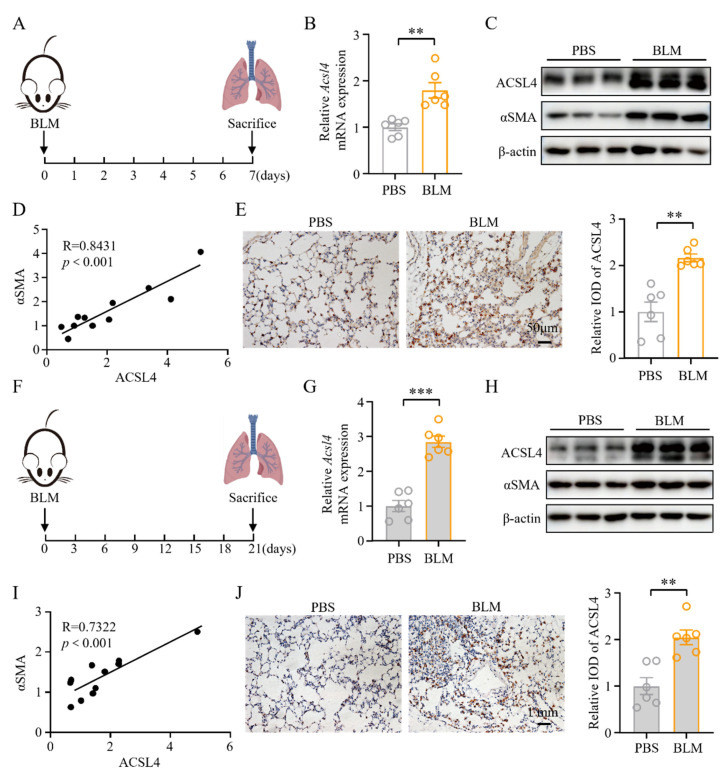
Acyl-CoA synthetase long-chain family member 4 (ACSL4) expression was increased in fibrotic lung tissues: (**A**) Scheme for the bleomycin (BLM)-induced lung fibrosis. Mice were euthanized on day 7 after BLM modeling. (**B**) The expression of *Acsl4* in lung tissues was quantified by qPCR; *n* = 6 for each group. (**C**) Western blot (WB) analysis of ACSL4 and α-smooth muscle actin (αSMA) in lung tissues. Representative bands are shown. (**D**) A correlation between ACSL4 protein and αSMA protein in lung tissues; *n* = 11. (**E**) Immunohistochemical staining of ACSL4 in lung tissues. Representative image and relative integrated optical density (IOD) analyses are shown. Original magnification: 200×; *n* = 6 for each group. (**F**) Scheme for the BLM-induced lung fibrosis. Mice were euthanized on day 21 after BLM modeling. (**G**) The expression of *Acsl4* in lung tissues was quantified by qPCR; *n* = 6 for each group. (**H**) WB analysis of ACSL4 and αSMA in lung tissues. Representative bands are shown. (**I**) A correlation between ACSL4 protein and αSMA protein in lung tissues; *n* = 11. (**J**) Immunohistochemical staining of ACSL4 in lung tissues. Representative images and relative IOD analyses are shown. Original magnification: 200×; *n* = 6 for each group. All data are the mean ± SEM; ** *p* < 0.01, *** *p* < 0.001 by Student’s *t*-test in panels (**B**,**E**,**G**,**J**). Correlation was assessed by Pearson correlation coefficient in panels (**D**,**I**).

On day 7 post-BLM modeling, the lung tissue was primarily in the acute inflammatory stage with early signs of fibrosis [30] (Figure 1A). More than 20% of the lung tissue showed consolidation (Figure S1A), along with significantly increased cellularity, alveolitis scores, and Ashcroft scores (Figure S1B–D). Collagen deposition was also markedly elevated in the model group compared to controls (Figure S1E–G).

ACSL4 is a key enzyme that catalyzes long-chain fatty acids [8], but its role in IPF remains unclear. To explore the involvement of ACSL4 in pulmonary fibrosis, we assessed its expression at this stage. Both mRNA and protein levels of ACSL4 were significantly upregulated in fibrotic lungs, accompanied by increased αSMA expression (Figure 1B,C and Figure S1H). A strong positive correlation was observed between ACSL4 and αSMA protein levels (R = 0.8431, *p* < 0.001; Figure 1D), and immunohistochemistry further confirmed elevated ACSL4 expression (Figure 1E).

By day 21, the lungs had progressed to the chronic fibrotic stage [30], with severe alveolar destruction and >80% consolidation (Figure 1F and Figure S1I). At this time point, lung tissues exhibited persistent cellular infiltration, high alveolitis and Ashcroft scores, and pronounced collagen accumulation, indicating sustained fibrotic severity (Figure S1J–O). The expression of *Acsl4* remained significantly higher in the model group compared to controls (Figure 1G). Correspondingly, the protein levels of ACSL4 and αSMA were significantly elevated in the model group (Figure 1H and Figure S1P), with a strong positive correlation observed between their expressions (R = 0.7322, *p* < 0.001) (Figure 1I). Immunohistochemistry further confirmed the increased expression of ACSL4 in fibrotic lung tissue (Figure 1J).

### 3.2. Pharmacological Inhibition of ACSL4 Alleviated Pulmonary Inflammation and Fibrosis in the Early Fibrotic Stage

**Figure 2:**
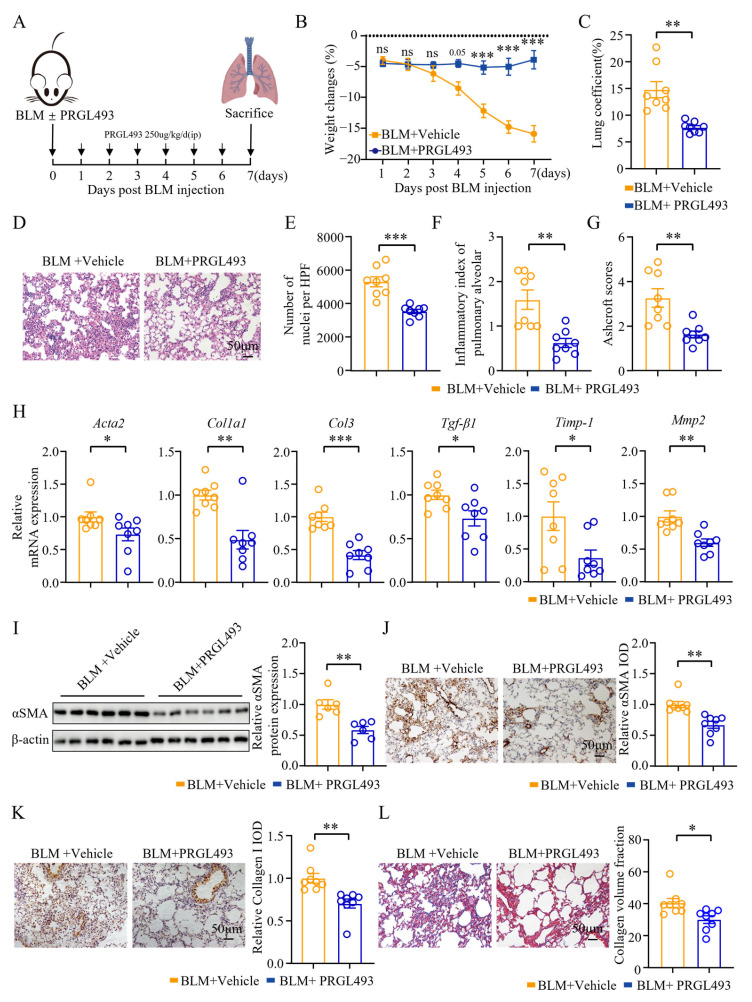
Pharmacological inhibition of ACSL4 alleviated pulmonary inflammation and fibrosis in the early fibrotic stage: (**A**) Scheme for BLM-induced lung fibrosis. The mice were treated with PRGL493 (250 μg/kg) or vehicle 1 h before BLM exposure on day 0, and then once a day. Mice were euthanized on day 7 after BLM modeling. (**B**) Weight changes of the mice; n = 8 for each group. (**C**) Lung coefficients of the mice; *n* = 8 for each group. (**D**) Hematoxylin–eosin (H&E) staining of lung tissues. Representative images are shown. Original magnification: 200×. (**E**) The number of nuclei per high-power field of lung tissues; *n* = 8 for each group. (**F**) Inflammatory index of the alveoli; *n* = 8 for each group. (**G**) The Ashcroft score; *n* = 8 for each group. (**H**) The expression of *Acta2*, *Col1a1*, *Col3*, *Tgf-β1*, tissue inhibitors of metalloproteinases-1 (*Timp1*), and matrix metalloproteinases-2 (*Mmp-2*) in lung tissues was quantified by qPCR; *n* = 8 for each group. (**I**) The expression of αSMA in the lung tissues was measured by WB. Representative bands and statistical plots are shown; *n* = 6 for each group. (**J**) Immunohistochemical staining of αSMA in lung tissues. Representative images and relative IOD analyses are shown. Original magnification: 200×; *n* = 8 for each group. (**K**) Immunohistochemical staining of collagen I in the lung tissues. Representative images and relative IOD analyses are shown. Original magnification: 200×; *n* = 8 for each group. (**L**) Masson staining of the lung tissues. Representative images and collagen volume fraction analyses are shown. Original magnification: 200×; *n* = 8 for each group. Data are the mean ± SEM; * *p* < 0.05, ** *p* < 0.01, *** *p* < 0.001 by two-way ANOVA followed by adjustments for multiple comparisons in panel (**B**), or Student’s *t*-test in panels (**C**,**E**–**L**); ns = not significant.

To further explore the function of ACSL4 in the early stage of pulmonary fibrosis, we utilized PRGL493, a potent and selective ACSL4 inhibitor (Figure 2A). The effects of PRGL493 were evaluated on day 7 post-modeling. We found that PRGL493 significantly alleviated BLM-induced weight loss (Figure 2B). Compared to the model group, PRGL493 also exhibited significant attenuation of pulmonary edema (Figure 2C). H&E staining revealed that PRGL493 mitigated alveolar septal thickening, preserved alveolar architecture, and reduced lung tissue consolidation (Figure 2D). Moreover, PRGL493 could decrease the number of infiltrating cells in lung tissues, as well as the alveolitis score and the Ashcroft score (Figure 2E–G).

Fibroblasts are key participants in lung tissue injury, and they can differentiate into myofibroblasts during lung tissue injury. Activated myofibroblasts produce key pro-fibrotic molecules such as αSMA, collagen, TGF-β, tissue inhibitors of metalloproteinases-1 (TIMP-1), and matrix metalloproteinases (MMPs) [31,32,33]. *Acta2*, *Col1a1*, and *Col3* are genes encoding αSMA, collagen type I, and collagen type III, respectively. After PRGL493 treatment, the mRNA expression of *Acta2*, *Col1a1*, *Col3*, *Tgf-β1*, *Timp1*, and *Mmp2* in the lung tissue was significantly decreased compared to the model group (Figure 2H). PRGL493 also reduced αSMA protein expression in lung tissues (Figure 2I,J). Collagen is a major component of ECM, and collagen types I, III, and V are overexpressed in fibrotic diseases [34,35,36].

To evaluate collagen deposition in lung tissue, we examined collagen I expression via immunohistochemistry. On day 7 post-modeling, the collagen I levels in the PRGL493-treated group were markedly lower than those in the control group (Figure 2K). Masson staining further confirmed these findings (Figure 2L).

In addition to its anti-fibrotic effects, PRGL493 also attenuated pulmonary inflammation, as evidenced by reduced IL-1β and IL-6 expression and decreased T-cell infiltration in lung tissues (Figure S2A,B). However, systemic inflammation remained unaffected, as no significant differences were observed in the peripheral blood levels of IL-1β, IL-2, IL-4, IL-6, IL-17, or TNFα between the model and PRGL493-treated groups (Figure S2C).

### 3.3. Pharmacological Inhibition of ACSL4 Attenuated Pulmonary Inflammation and Fibrosis in the Chronic Stage

**Figure 3:**
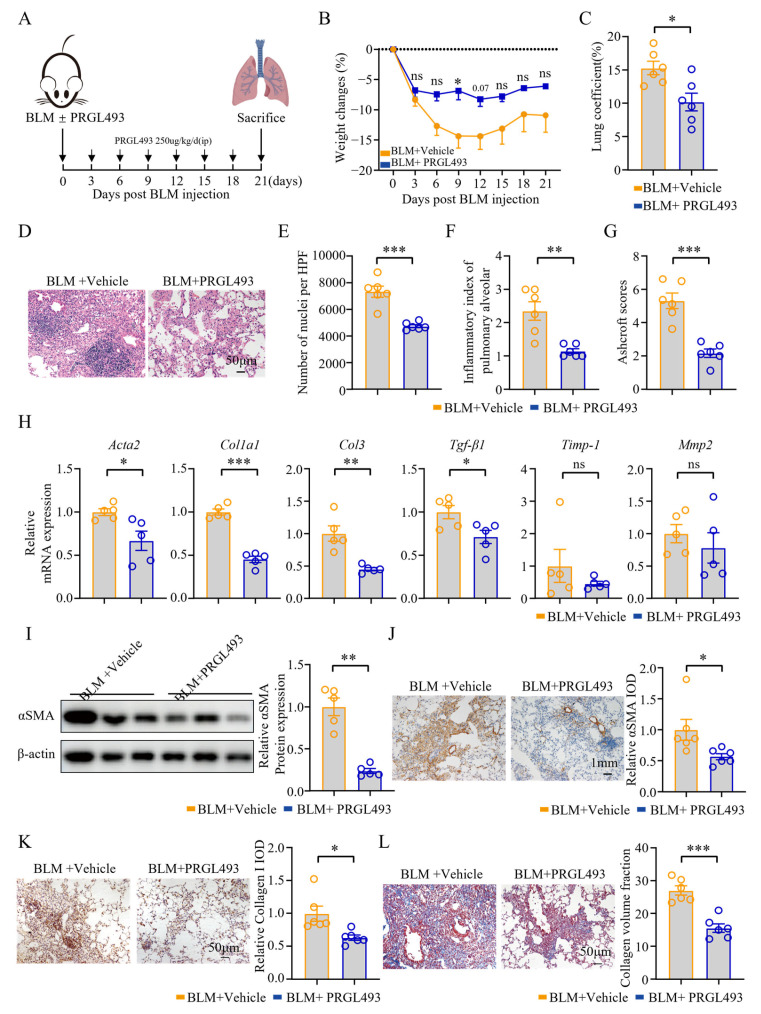
Pharmacological inhibition of ACSL4 alleviated pulmonary inflammation and fibrosis in the chronic fibrotic stage. (**A**) Scheme for BLM-induced lung fibrosis. The mice were treated with PRGL493 (250 μg/kg) or vehicle 1h before BLM exposure on day 0, and then once a day. Mice were euthanized on day 21 after BLM modeling. (**B**) Weight changes of the mice; *n* = 6 for each group. (**C**) Lung coefficients of the mice; *n* = 6 for each group. (**D**) H&E staining of the lung tissues. Representative images are shown. Original magnification: 200×. (**E**) Number of nuclei per high-power field of the lung tissues; *n* = 6 for each group. (**F**) Inflammatory index of the alveoli; *n* = 6 for each group. (**G**) The Ashcroft score; *n* = 6 for each group. (**H**) The expression of *Acta2*, *Col1a1*, *Col3*, *Tgf-β1*, *Timp-1*, and *Mmp-2* in lung tissues was quantified by qPCR; *n* = 5 for each group. (**I**) WB analysis of αSMA in lung tissues. Representative bands and statistical plots are shown; *n* = 5 for each group. (**J**) Immunohistochemical staining of αSMA in lung tissues. Representative images and relative IOD analyses are shown. Original magnification: 200×; *n* = 6 for each group. (**K**) Immunohistochemical staining of collagen I in lung tissues. Representative images and relative IOD analyses are shown. Original magnification: 200×; *n* = 6 for each group. (**L**) The Masson staining of the lung tissues. Representative images and collagen volume fraction analyses are shown. Original magnification: 200×; *n* = 6 for each group. Data are the mean ± SEM; * *p* < 0.05, ** *p* < 0.01, *** *p* < 0.001 by two-way ANOVA followed by adjustments for multiple comparisons in panel (**B**), or Student’s *t*-test in panels (**C**,**E**–**L**). ns = not significant.

To explore the impact of ACSL4 on chronic fibrosis, mice were evaluated on day 21 post-BLM administration (Figure 3A). PRGL493 effectively attenuated BLM-induced weight loss, pulmonary edema, and pathological damage (Figure 3B–D). Additionally, PRGL493 reduced the number of cells in lung tissue (Figure 3E) and significantly decreased the alveolitis and Ashcroft scores (Figure 3F,G). In the PRGL493-treated group, the expression of *Acta2*, *Col1A1*, *Col3*, and *Tgf-β1* in lung tissue was significantly decreased compared to the model group (Figure 3H). Unlike in the 7-day model, no significant differences in the expression of *Timp1* and *Mmp2* were observed between the PRGL493-treated and model groups (Figure 3H). PRGL493 also suppressed αSMA protein expression (Figure 3I,J) and reduced collagen deposition in the lungs (Figure 3K,L).

On day 21 post-BLM administration, PRGL493 significantly reduced Il-6 expression in lung tissues and peripheral blood, as well as T-cell infiltration in lung tissues (Figure S2D–F). There were no differences in IL-1β and TNF-α between the PRGL493 group and the model group (Figure S2D). PRGL493 did not affect the levels of IL-1β, IL-2, IL-4, IL-17, or TNFα in peripheral blood (Figure S2F).

### 3.4. ACSL4 Promoted Activation and Migration but Not Proliferation of Lung Fibroblasts

**Figure 4:**
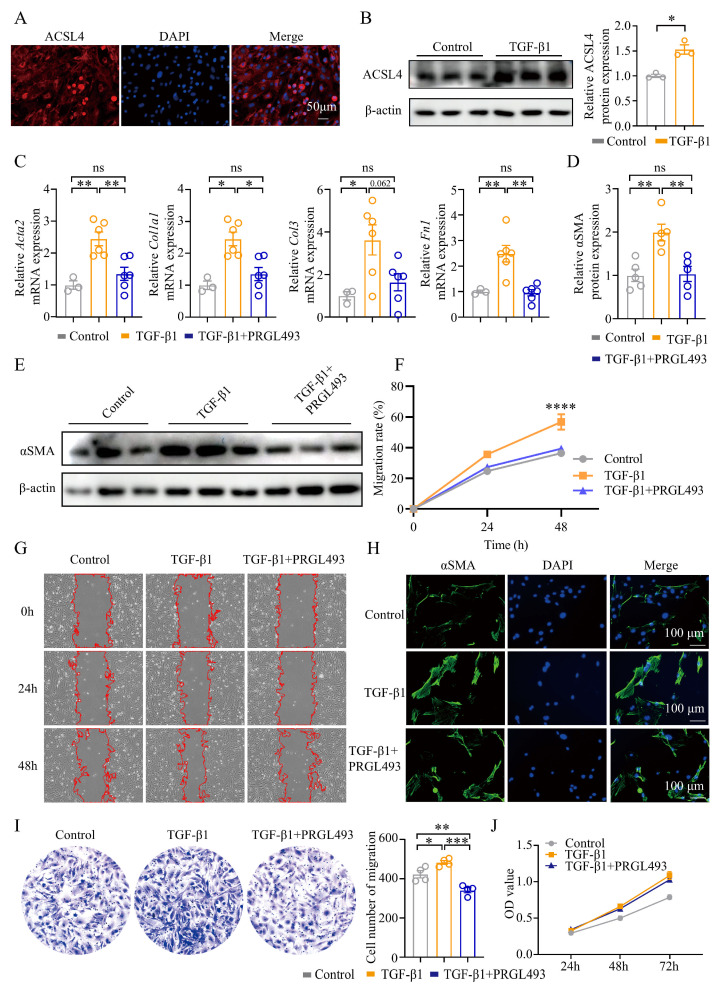
Blocking ACSL4 suppressed the activation and migration of lung fibroblasts: (**A**) The expression of ACSL4 in lung fibroblasts. Representative immunofluorescence images are shown: ACSL4 (red) and DAPI (blue). Original magnification: 100×. (**B**) WB analysis of ACSL4 in lung fibroblasts treated with PBS control or TGF-β1 (5 ng/mL). Representative bands and statistical plots are shown; *n* = 3 for each group. (**C**) The expression of *Acta2*, *Col1a1*, *Col3*, and *Fn1* was quantified by qPCR; *n* = 3–6 for each group. (**D**,**E**) WB analysis of αSMA in the lung fibroblasts treated with different treatments. Representative bands and statistical plots are shown; *n* = 5 for each group. (**F**,**G**) Lung fibroblasts’ migration after different treatments was assessed by the cell scratch assay. Representative images and statistical analysis are shown; *n* = 4 for each group. (**H**) The expression of αSMA protein in the lung fibroblasts treated with different treatments was tested by immunofluorescence staining. Representative immunofluorescence plots are shown: αSMA (green) and DAPI (blue). Original magnification: 200×. (**I**) The spatial migration ability of lung fibroblasts was tested by the transwell assay. Representative plot and statistical plot are shown; *n* = 4 for each group. (**J**) The proliferation of the lung fibroblasts was tested by CCK8 assay. Data are the mean ± SEM; * *p* < 0.05, ** *p* < 0.01, *** *p* < 0.001, **** *p* < 0.0001 by Student’s *t*-test in panel (**B**), one-way ANOVA followed by adjustments for multiple comparisons in panels (**C**,**D**,**I**), or two-way ANOVA followed by adjustments for multiple comparisons in panels (**F**,**J**); ns = not significant.

ACSL4 is expressed in multiple organs, such as the kidneys, pancreas, and nervous system [37,38], but its expression in lung fibroblasts has not been defined. To confirm the expression of ACSL4 in lung fibroblasts, we used vimentin as a fibroblast marker and assessed ACSL4 by immunofluorescence. We found that the purity of the extracted lung fibroblasts exceeded 95% (Figure S3A). We also found that the mouse lung fibroblasts expressed ACSL4, predominantly localized in the cytoplasm and especially enriched in the perinuclear region (Figure 4A).

We used TGF-β1 to induce fibroblast activation, as described in previous studies [39]. The qPCR results confirmed that stimulation with 5 or 10 ng/mL TGF-β1 for 24 or 48 h could induce the expression of *Acta2* and *Col1a1* in fibroblasts (Figure S3B,C). We selected 5 ng/mL TGF-β1 for 48 h as the activation condition for lung fibroblasts. Under these conditions, both *Acsl4* mRNA and ACSL4 protein expression were significantly increased compared to controls (Figures S3D and 4B).

To investigate the role of ACSL4 in lung fibroblasts, cells were treated with PRGL493. Treatment with 10 nM PRGL493 for 24, 48, or 72 h did not affect fibroblast viability (Figure S3E). Fibronectin 1 (*Fn1*), a key ECM component in pulmonary fibrosis, was upregulated upon TGF-β1 stimulation, along with *Acta2*, *Col1a1*, and *Col3*. PRGL493 significantly attenuated the expression of *Fn1*, *Acta2*, and *Col1a1*, and it showed a trend toward reducing *Col3* expression (Figure 4C). Consistently, PRGL493 reduced αSMA protein levels induced by TGF-β1 (Figure 4D,E,H).

Fibroblast migration, a hallmark of pulmonary fibrosis progression [40], was significantly enhanced following TGF-β1 treatment for 24 and 48 h. PRGL493 treatment effectively counteracted this effect, reducing fibroblast migration in both the horizontal wound-healing assay (Figure 4F,G) and the transwell migration assay (Figure 4I). Fibroblast proliferation is essential to the pathogenesis of fibrosis [41]. Notably, while TGF-β1 significantly increased lung fibroblast proliferation, PRGL493 had no significant effect (Figure 4J).

### 3.5. Inhibition of C5a/C5aR1 Signaling Reduced the Expression of ACSL4 in the Early Stage of Pulmonary Fibrosis

**Figure 5:**
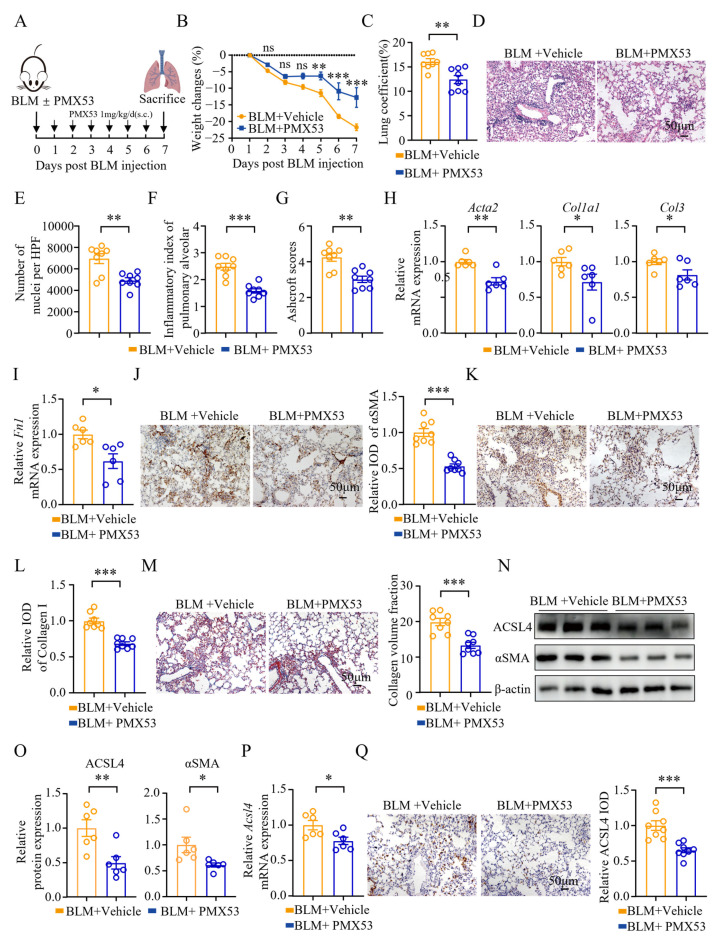
Effects of blocking the C5a/C5aR1 signaling on the early stage of lung fibrosis: (**A**) Scheme for the BLM-induced lung fibrosis. The mice were treated with PMX53 (1 mg/kg) or vehicle 1 h before BLM exposure on day 0, and then once a day. Mice were euthanized on day 7 after BLM modeling. (**B**) Weight changes of the mice; *n* = 8 for each group. (**C**) Lung coefficients of the mice; n = 8 for each group. (**D**) H&E staining of the lung tissues. Representative images are shown. Original magnification: 200×. (**E**) The number of nuclei per high-power field of lung tissues; *n* = 8 for each group. (**F**) The inflammatory index of the alveoli; *n* = 8 for each group. (**G**) The Ashcroft score; *n* = 8 for each group. (**H**,**I**) The expression of *Acta2*, *Col1a1*, *Col3*, and *Fn1* in lung tissues was quantified by qPCR; *n* = 6 for each group. (**J**) The expression of αSMA protein in lung tissues. Representative immunohistochemical plots and statistical plots are shown. Original magnification: 200×; *n* = 8 for each group. (**K**,**L**) Immunohistochemical staining of collagen I in lung tissues. Representative images and relative IOD analyses are shown. Original magnification: 200×; *n* = 8 for each group. (**M**) Masson staining of the lung tissues. Representative images and collagen volume fraction analyses are shown. Original magnification: 200×; *n* = 8 for each group. (**N**,**O**) WB analysis of the ACSL4 and αSMA protein in lung tissues. Representative bands and statistical plots are shown; *n* = 6 for each group. (**P**) The expression of *Acsl4* in lung tissues was quantified by qPCR; *n* = 6 for each group. (**Q**) Immunohistochemical staining of ACSL4 protein in the lung tissues. Representative images and relative IOD analyses are shown; *n* = 8 for each group. Data are the mean ± SEM; * *p* < 0.05, ** *p* < 0.01, *** *p* < 0.001 by two-way ANOVA followed by adjustments for multiple comparisons in panel (**B**), or Student’s *t*-test in panels (**C**,**E**–**J**,**L**,**M**,**O**–**Q**); ns = not significant.

C5a/C5aR1 signaling is recognized as a key mediator of inflammation and fibrosis in pulmonary fibrosis. To investigate whether this pathway regulates the expression of ACSL4 during the early stage of fibrosis, we employed the C5aR1 antagonist PMX53. PMX53 was administered 1 h before fibrosis induction to evaluate whether preemptive blockade of C5a/C5aR1 signaling could mitigate fibrotic progression. Lung tissues were collected on day 7 post-modeling, as illustrated in Figure 5A. PMX53 treatment effectively attenuated BLM-induced weight loss (Figure 5B) and pulmonary edema (Figure 5C).

Histopathological analysis revealed that PMX53 ameliorated BLM-induced lung damage (Figure 5D). Compared to the model group, PMX53 significantly reduced cell infiltration in the lung tissue, as well as the alveolitis scores and the Ashcroft scores (Figure 5E–G).

Blockade of the C5a/C5aR1 signaling markedly decreased the expression of fibrosis-related genes, including *Acta2*, *Col1a1*, *Col3*, and *Fn1*, in the early stage of fibrosis (Figure 5H,I). Additionally, PMX53 inhibited TGF-β1-induced expression of αSMA protein and reduced the deposition of collagen I, which was further confirmed by Masson staining (Figure 5J–M). Notably, the blockade of C5a/C5aR1 signaling also decreased the expression of ACSL4 protein and *Acsl4* mRNA (Figure 5N–Q).

### 3.6. Inhibition of C5a/C5aR1 Signaling Reduced the Expression of ACSL4 in Chronic Pulmonary Fibrosis

**Figure 6:**
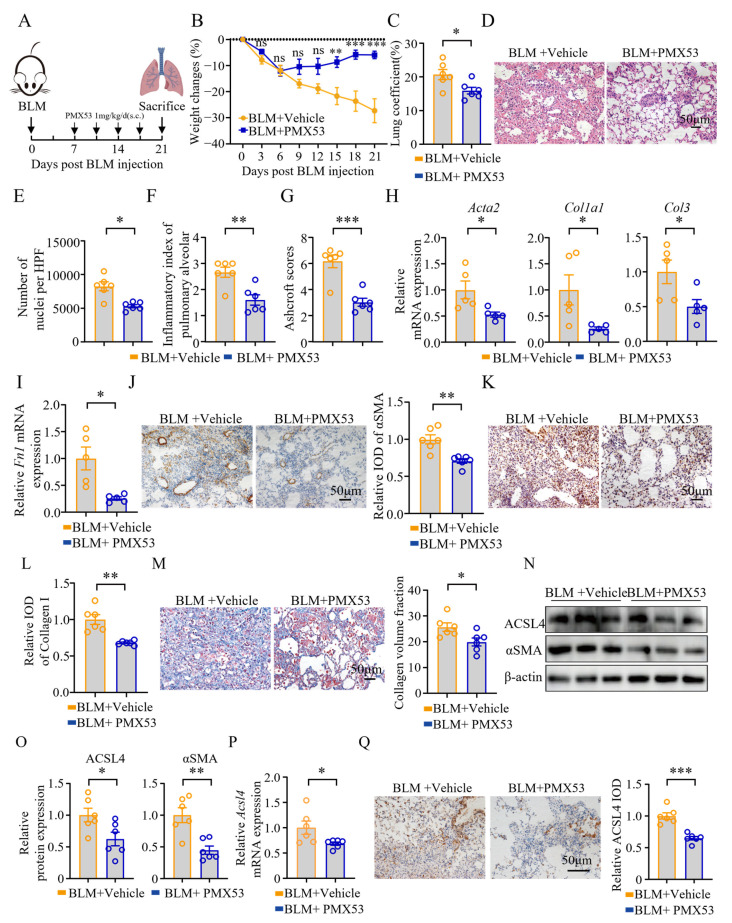
Inhibition of C5a/C5aR1 signaling reduced ACSL4 levels and attenuated lung inflammation and fibrosis in the chronic stage of pulmonary fibrosis: (**A**) Scheme of BLM-induced lung fibrosis. Mice were euthanized on day 21 after BLM modeling. (**B**) Weight changes of the mice; *n* = 6 for each group. (**C**) Lung coefficients of the mice; *n* = 6 for each group. (**D**) H&E staining of the lung tissues. Representative images are shown. Original magnification: 200×. (**E**) Number of nuclei per high-power field of the lung tissues; *n* = 6 for each group. (**F**) The inflammatory index of the alveoli; *n* = 6 for each group. (**G**) The Ashcroft score; *n* = 6 for each group. (**H**,**I**) The expression of *Acta2*, *Col1a1*, *Col3*, and *Fn1* in lung tissues was quantified by qPCR; *n* = 5 for each group. (**J**) Immunohistochemical staining of αSMA in lung tissues. Representative images and relative IOD analyses are shown. Original magnification: 200×; *n* = 6 for each group. (**K**,**L**) Immunohistochemical staining of collagen I in lung tissues. Representative images and relative IOD analyses are shown. Original magnification: 200×; *n* = 6 for each group. (**M**) Masson staining of lung tissues. Representative images and collagen volume fraction analyses are shown. Original magnification: 200×; *n* = 6 for each group. (**N**,**O**) WB analysis of the ACSL4 and αSMA proteins in lung tissues. Representative bands and statistical plots are shown; *n* = 6 for each group. (**P**) The expression of *Acsl4* in lung tissues was quantified by qPCR; *n* = 6 for each group. (**Q**) Immunohistochemical staining of ACSL4 in lung tissues. Representative images and relative IOD analyses are shown; *n* = 6 for each group. Data are the mean ± SEM; * *p* < 0.05, ** *p* < 0.01, *** *p* < 0.001 by two-way ANOVA followed by adjustments for multiple comparisons in panel (**B**), or Student’s *t*-test in panels (**C**,**E**–**J**,**L**,**M**,**O**–**Q**); ns = not significant.

To investigate the therapeutic potential of C5a/C5aR1 signaling in established pulmonary fibrosis, we used PMX53 once a day from day 7 post-modeling. The samples were collected on day 21 post-modeling, as illustrated in Figure 6A. The results demonstrated that blocking C5a/C5aR1 signaling after fibrosis had been initiated still effectively inhibited BLM-induced weight loss (Figure 6B) and attenuated pulmonary edema (Figure 6C). Additionally, histological analysis revealed that PMX53 treatment reduced the number of cells in the lung tissue and significantly alleviated alveolitis and fibrosis, as evidenced by the lower alveolitis scores and the Ashcroft scores in the PMX53 group (Figure 6E–G).

The blockade of the C5a/C5aR1 signaling can reduce the expression of *Acta2*, *Col1a1*, *Col3*, and *Fn1* (Figure 6H,I). Furthermore, PMX53 reduced the expression of αSMA protein (Figure 6J,N,O) and the deposition of collagen (Figure 6K–M), which was confirmed through WB and immunohistochemistry. Notably, the expression of ACSL4, both in mRNA (Figure 6P) and protein (Figure 6N,O,Q), was significantly diminished after being treated with PMX53.

### 3.7. Blocking ACSL4 Reduced Lung Fibroblast Activation and Migration Induced by the C5a/C5aR1 Signaling

**Figure 7:**
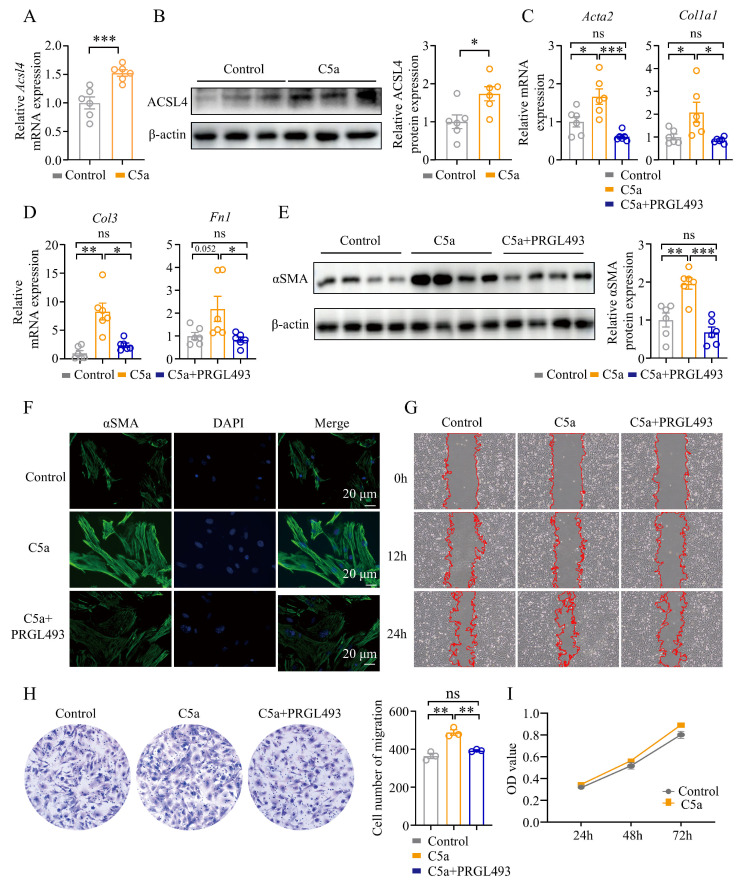
Blocking ACSL4 reduced C5a/C5aR1 signaling-induced activation and migration of lung fibroblasts: (**A**) The expression of *Acsl4* was quantified by qPCR; *n* = 6 for each group. (**B**) WB analysis of ACSL4 in lung fibroblasts. Representative bands and statistical plots are shown; *n* = 6 for each group. (**C**,**D**) The expression of *Acta2*, *Col1a1*, *Col3*, and *Fn1* was quantified by qPCR; *n* = 6 for each group. (**E**) WB analysis of αSMA in lung fibroblasts. Representative bands and statistical plots are shown; *n* = 6 for each group. (**F**) The expression of αSMA was tested by immunofluorescence. Representative plots are shown. Original magnification: 400×; αSMA (green) and DAPI (blue). (**G**) The cell scratch assay was used to assess lung fibroblasts’ migration. Representative images are shown. (**H**) The transwell assay. Representative plot and statistical plots of the transwell assay results are shown; *n* = 3 for each group. (**I**) The proliferation of fibroblasts was tested by CCK8. Data are the mean ± SEM; * *p* < 0.05, ** *p* < 0.01, *** *p* < 0.001 by Student’s *t*-test in panels (**A**,**B**), one-way ANOVA followed by adjustments for multiple comparisons in panels (**C**–**E**,**H**), or two-way ANOVA followed by adjustments for multiple comparisons in panel (**I**); ns = not significant.

Lung fibroblasts expressed C5aR1 (CD88) (Figure S4A). C5a induced the activation of pulmonary fibroblasts in a time- and concentration-dependent manner (Figure S4B). The results showed that C5a could increase the expression of ACSL4 in lung fibroblasts (Figure 7A,B). We also found that inhibiting ACSL4 with PRGL493 in the presence of C5a reduced the expression of fibrosis-related genes, including *Acta2*, *Col1a1*, *Col3*, and *Fn1* (Figure 7C,D), and decreased the expression of αSMA protein (Figure 7D,F). Additionally, PRGL493 inhibited C5a-induced fibroblast migration, both in horizontal migration (Figure 7G and Figure S4C) and spatial migration (Figure 7H). Notably, C5a did not affect fibroblasts’ proliferation (Figure 7I).

### 3.8. Blockade of Calcium Signaling Attenuated ACSL4 Expression Induced by the C5a/C5aR1 Signaling

**Figure 8:**
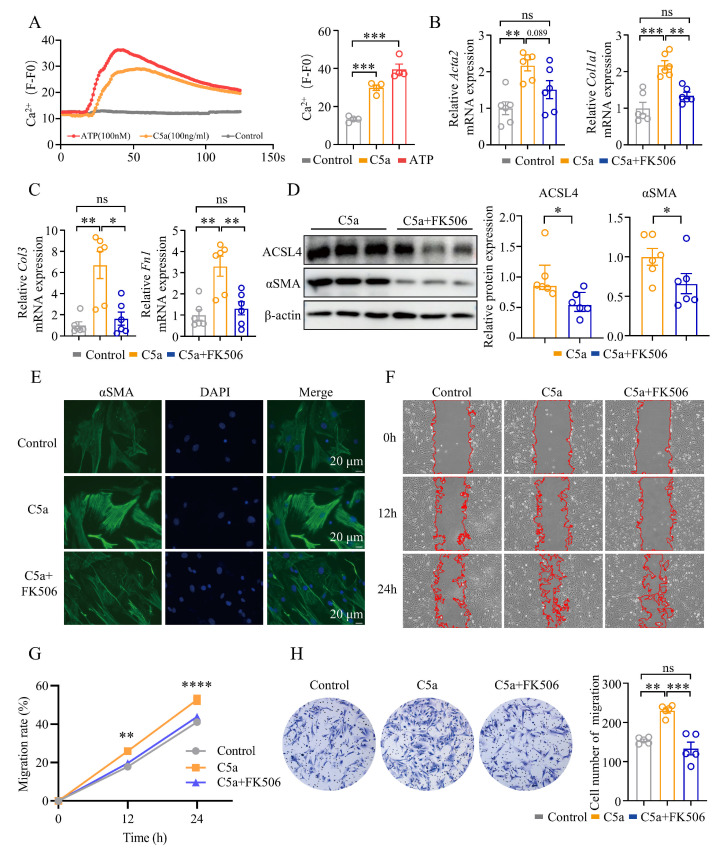
Blockade of calcium signaling attenuated ACSL4 expression induced by the C5a/C5aR1 signaling: (**A**) The calcium concentration of lung fibroblasts was tested by calcium imaging. Representative plots and statistical plots are shown; *n* = 4 for each group. (**B**,**C**) The expression of *Acta2*, *Col1a1*, *Col3*, and *Fn1* was quantified by qPCR; *n* = 6 for each group. (**D**) WB analysis of ACSL4 and αSMA in lung fibroblasts. Representative bands and statistical plots are shown; *n* = 6 for each group. (**E**) The expression of αSMA was tested by immunofluorescence. Plots are shown. Original magnification: 400×; αSMA (green) and DAPI (blue). (**F**,**G**) The horizontal migration ability of lung fibroblasts was tested by the cell scratch assay. Representative plots and statistical plots are shown; *n* = 4 for each group. (**H**) The spatial migration ability of lung fibroblasts was tested by transwell assay. Representative plot and statistical plot are shown; *n* = 5 for each group. Data are the mean ± SEM; * *p* < 0.05, ** *p* < 0.01, *** *p* < 0.001, , **** *p* < 0.0001 by one-way ANOVA followed by adjustments for multiple comparisons in panels (**A**–**C**,**H**), Student’s *t*-test in panel (**D**), or two-way ANOVA followed by adjustments for multiple comparisons in panel (**G**); ns = not significant.

Previous studies have demonstrated that TGF-β1 treatment elevates the calcium ion concentration in lung fibroblasts [42]. However, the impact of C5a on calcium ion dynamics in fibroblasts remains unexplored. In the present study, we used calcium imaging to investigate the influence of C5a on the calcium ion concentration in lung fibroblasts. Our findings demonstrated that treatment with 100 ng/mL C5a significantly increased the intracellular calcium ion concentration in lung fibroblasts (Figure 8A). To determine whether calcium signaling acts as a molecular switch for the expression of ACSL4, we employed FK506, a calcineurin pathway inhibitor, to block downstream calcium signaling. Preliminary experiments confirmed that using FK506 (1, 5, or 10 μM) for 12, 24, or 48 h did not affect the fibroblasts’ viability (Figure S5A), leading us to select 1 μM FK506 for subsequent assays.

To elucidate the role of C5a in the calcium signaling of fibroblasts, the fibroblasts were co-treated with C5a and FK506. FK506 downregulated the expression of fibrosis-related genes induced by C5a, including *Col1a1*, *Col3*, and *Fn1*, with a trend toward reduction in *Acta2* expression (Figure 8B,C). At the protein level, FK506 effectively reduced C5a-induced expression of αSMA and ACSL4 (Figure 8D,E and Figure S5B). Furthermore, the horizontal and spatial migration capacities were significantly impaired in cells treated with C5a plus FK506 compared to C5a alone (Figure 8F–H).

## 4. Discussion

In the present study, we established a pulmonary fibrosis model through a single intratracheal BLM administration, which progressed the acute inflammatory phase for one week, followed by gradual fibrosis development and eventual subsidence 3–4 weeks post-modeling [30,43]. We selected two time points to observe, day 7 and day 21, to assess the role of ACSL4 in different stages of pulmonary fibrosis. The 7-day model allowed us to examine ACSL4′s impact on the inflammatory and early fibrosis stages, while the 21-day model provided insights into its role during the chronic fibrosis stage. Our findings revealed that the expression of ACSL4 was elevated in both the 7-day and 21-day models compared to the control groups, suggesting a role for ACSL4 in the initiation and progression of pulmonary fibrosis.

We used PRGL493 to inhibit ACSL4 to investigate the function of ACSL4. We found that PRGL493 could alleviate BLM-induced weight loss, pulmonary edema, alveolitis scores, fibrosis scores, and collagen deposition in the lung tissue in the 7-day model and 21-day model. Notably, in the 7-day model, PRGL493 could reduce the expression of *Acta2*, *Col1a1*, *Col3*, *Tgf-β1*, *Timp1*, and *Mmp2*, suggesting that ACSL4 influences the production and degradation of collagen during the early stages of fibrosis. In the 21-day model, PRGL493 could not influence the expression of *Timp1* and *Mmp2*, but it could reduce the expression of *Acta2*, *Tgf-β1*, *Col1a1*, and *Col3*. While *Acta2*, *Tgf-β1*, *Col1a1*, and *Col3* are associated with the activation of fibroblasts and production of collagen [44,45], *Mmp-2* and *Timp-1* are related to the decomposition of collagen [46,47]. Thus, it is believed that ACSL4 affects both the production and decomposition of collagen in the early stage of fibrosis, while it mainly takes part in the production of collagen in the chronic stage. These findings confirm that ACSL4 is a crucial regulator of pulmonary fibrosis in both the inflammatory phase and fibrotic processes.

The activation, migration, and proliferation of fibroblasts are essential in the development of fibrosis [48,49,50]. Our results demonstrated that ACSL4 was upregulated in lung fibroblasts upon TGF-β1 stimulation. Inhibition of ACSL4 with PRGL493 reduced the expression of *Acta2*, *Col1a1*, *Col3*, and *Fn1* induced by TGF-β1, supporting the role of ACSL4 in the activation of fibroblasts. Additionally, PRGL493 inhibited the migration of lung fibroblasts induced by TGF-β1. The migration of lung fibroblasts is affected by many factors, including ECM-derived mechanical force, microgravity effects, the YAP protein, and the F-actin protein [51,52]. A study on renal fibrosis revealed that, after stimulating human renal tubular epithelial cells with calcium oxalate crystals, activated YAP could be translocated to the nucleus to increase the expression of ACSL4 and to aggravate their fibrotic phenotype [14]. We also found that PRGL493 did not impact the proliferation of fibroblasts induced by TGF-β1.

The C5a/C5aR1 signaling has been implicated in the pathogenesis of pulmonary fibrosis, and interactions between C5a and TGF-β signaling have been suggested to mediate epithelial injury in lung fibrosis [53]. One study established a model of pulmonary fibrosis by overexpressing TGF-β in the trachea, and the C5a receptor was blocked 2 weeks after modeling. On day 28 after modeling, local collagen deposition in the lung tissue was reduced, and the expression of Smad7 (a negative regulator of the TGF-β signaling) was restored [54]. The interaction between C5a and its receptor activates the TGF-β signaling, which may be one of the reasons for C5a-induced pulmonary fibrosis. This phenomenon has been reported in other fibrotic diseases, such as renal fibrosis caused by ischemia–reperfusion and diabetic nephropathy [55,56]. We hypothesized that ACSL4 might be the effector of both the C5a/C5aR1 signaling and the TGF-β1 signaling. To confirm this hypothesis, we blocked the C5a/C5aR1 signaling with PMX53 and found a significant reduction in the expression of ACSL4 in lung fibroblasts. Importantly, blocking the C5a/C5aR1 signaling either before or after the initiation of fibrosis led to significant reductions in lung tissue cell numbers, alveolitis scores, Ashcroft scores, collagen deposition, and the expression of fibrosis-related genes, including *Acta2*, *Col1a1*, *Col3*, and *Fn1*. These findings support the hypothesis that ACSL4 is a critical effector in the C5a/C5aR1-mediated activation of fibroblasts.

Notably, PMX53-treated mice exhibited more weight loss than those treated with PRGL493. This may reflect the broader immune-regulatory role of C5aR1, whose inhibition may impair not only pro-fibrotic but also protective inflammatory processes. In contrast, ACSL4 inhibition targets fibroblast activation more selectively, potentially leading to fewer systemic effects. This distinction underscores the therapeutic trade-offs between targeting upstream immune pathways and downstream fibrotic effectors.

Our findings suggest that ACSL4 functions downstream of C5aR1 in fibroblast activation. Therefore, ACSL4 inhibition may be sufficient to attenuate C5a-driven fibrosis. Although dual inhibition of C5aR1 and ACSL4 may theoretically provide additive effects, targeting both nodes within a linear pathway may yield limited additional benefit. Nonetheless, this possibility merits further investigation using genetic or pharmacological approaches.

Calcium signaling is a well-established regulator of fibroblast activation and migration [42]. We hypothesized that C5a/C5aR1 promotes ACSL4 expression via calcium signaling. Supporting this, we demonstrated that C5a could increase the concentration of calcium in lung fibroblasts. FK506, an inhibitor of calcium signaling, can reduce the expression of ACSL4 induced by C5a. These results suggest that the C5a/C5aR1 signaling promotes pulmonary fibrosis by stimulating ACSL4 expression via calcium signaling, which, in turn, enhances fibroblasts’ activation and migration.

This calcium-dependent mechanism aligns with several established findings: First, the calcium signaling pathway has been shown to mediate the expression of ECM genes induced by TGF-β1 through calmodulin-dependent protein kinase II [42]. Second, the connection of C5a and calcium has been established in various cell types, including epithelial cells [57], macrophages [58,59,60], and neutrophils [61]. However, C5a fails to induce calcium signaling in HeLa cells [62], which may be caused by the expression of C5a receptors in different cells. In lung fibroblasts, we confirmed the presence of C5aR1—the classical GPCR, known to mediate calcium signaling—as opposed to the non-classical inducible receptor C5aR2 [63]. This functional dichotomy holds critical importance in light of the extensively characterized GPCR–calcium signaling axis, wherein GPCRs act as both sensors and modulators of cytosolic calcium homeostasis through bidirectional crosstalk mechanisms [64,65]. Our experiments using FK506 provide direct evidence for this mechanism: inhibition of calcium signaling not only attenuated the expression of ACSL4 induced by C5a but also suppressed the activation and migration induced by C5a. These findings collectively support our hypothesis that calcium signaling serves as a crucial bridge connecting C5a/C5aR1 signaling to the upregulation of ACSL4 in pulmonary fibrosis, positioning ACSL4 as a key regulatory node integrating signals from both the C5a/C5aR1 and TGF-β signaling in the pathogenesis of fibrosis.

Taken together, our findings not only confirm a role for ACSL4 in pulmonary fibrosis but also uncover a previously unrecognized upstream pathway—C5a/C5aR1–calcium signaling—that regulates its expression in fibroblasts. Unlike prior studies that focused on ferroptosis or macrophages [15,66], we demonstrate a fibroblast-intrinsic, non-ferroptotic function of ACSL4 in promoting FMT. Moreover, targeting upstream C5aR1 may represent an alternative therapeutic strategy to modulate this pathway. These results expand our understanding of how immune signaling converges on downstream effectors like ACSL4 to drive fibroblast activation and tissue remodeling. Future studies should further elucidate the downstream signaling events mediated by ACSL4 in fibroblasts and validate the clinical relevance of the C5aR1–calcium–ACSL4 axis using patient-derived lung tissues.

## 5. Conclusions

Our study demonstrated that the C5a/C5aR1–calcium signaling is activated in lung fibroblasts via pulmonary fibrosis models and lung fibroblasts stimulated in vitro. Activation of this signaling contributes to the pathogenesis of pulmonary fibrosis by upregulating ACSL4 expression and promoting FMT. Therefore, targeting the C5a/C5aR1–calcium–ACSL4 axis could effectively ameliorate fibrotic progression by reducing fibroblast activation and migration, potentially offering a novel therapeutic strategy for pulmonary fibrosis treatment.

## Data Availability

The data used and/or analyzed during the current study are available from the corresponding author upon reasonable request.

## Supplementary Materials

The following supporting information can be downloaded at: https://www.mdpi.com/article/10.3390/biom15081106/s1, Figure S1. Pathological changes of lung tissue at early and chronic stage of pulmonary fibrosis; Figure S2. Expression of inflammatory factors in lung tissue and peripheral blood on day 7 and day 21 after bleomycin modeling; Figure S3. Identification of lung fibroblast, measurement of the concentration of TGF-β1 and PRGL493; Figure S4. The expression of CD88 in lung fibroblasts and induction of lung fibroblasts by C5a; Figure S5. The Effect of FK506 on cell viability and the expression of ACSL4; Supplemental Table S1. Primer for qPCR.
