# Supplementary material for: ACSL4 Drives C5a/C5aR1–Calcium-Induced Fibroblast-to-Myofibroblast Transition in a Bleomycin-Induced Mouse Model of Pulmonary Fibrosis
Source: Biomolecules. 2025 Jul 31;15(8):1106. doi: 10.3390/biom15081106 (PMC12383839; doi:10.3390/biom15081106)
Supplement: Supplementary file 1 [file biomolecules-15-01106-s001.zip › biomolecules-3689094-Origianl WB.pdf]

Figure.S1C

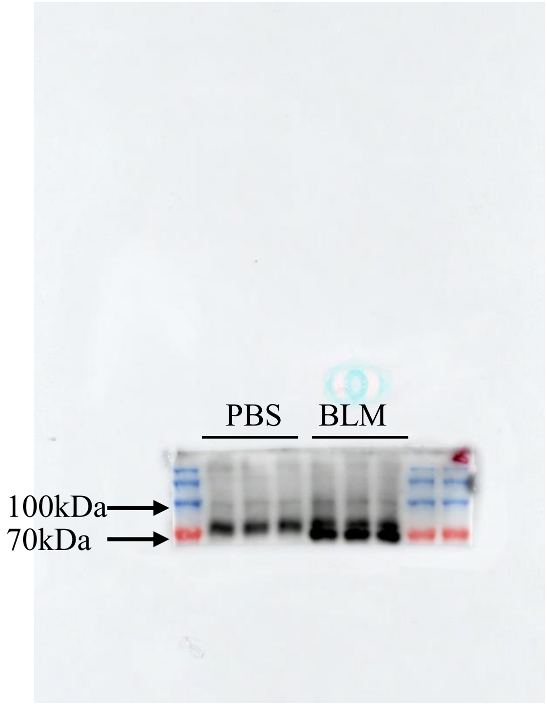

ACSL4

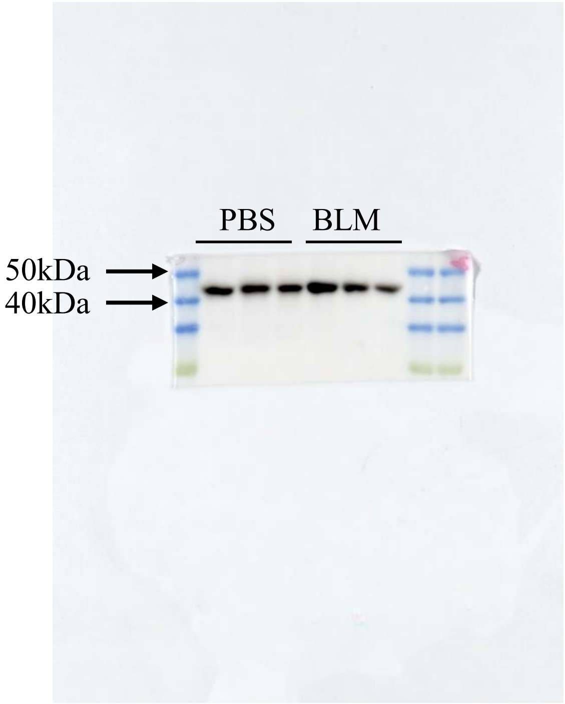

β-actin

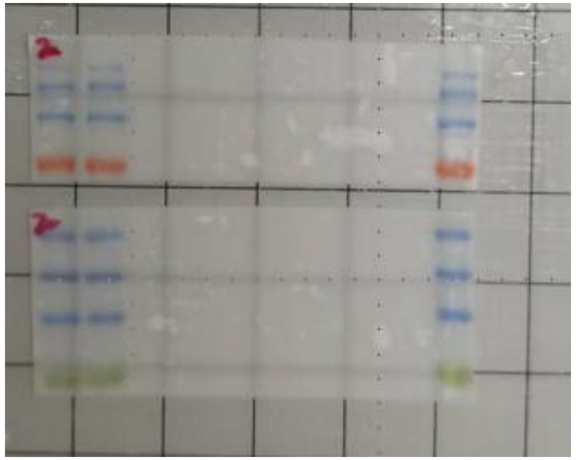

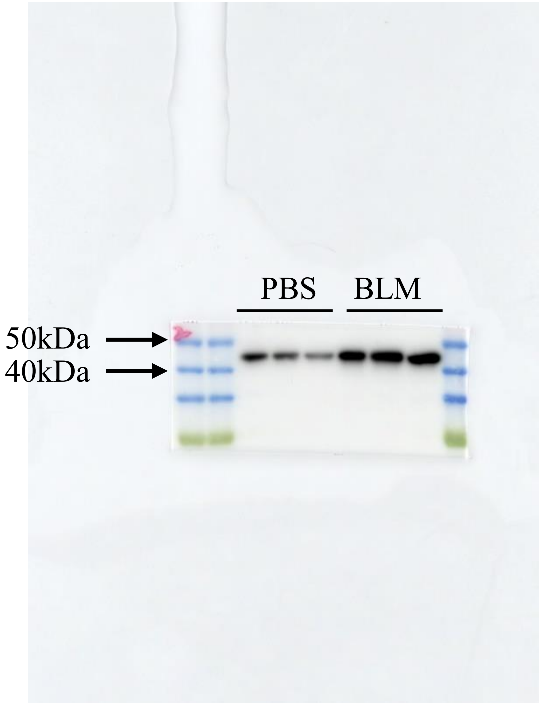

αSMA

Figure.S1H

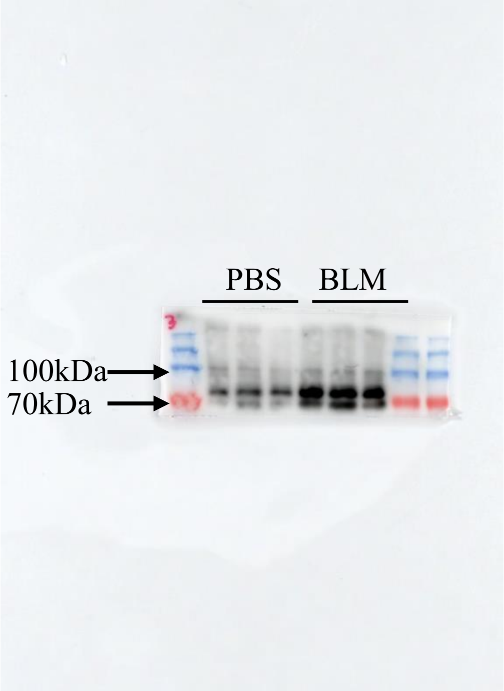

ACSL4

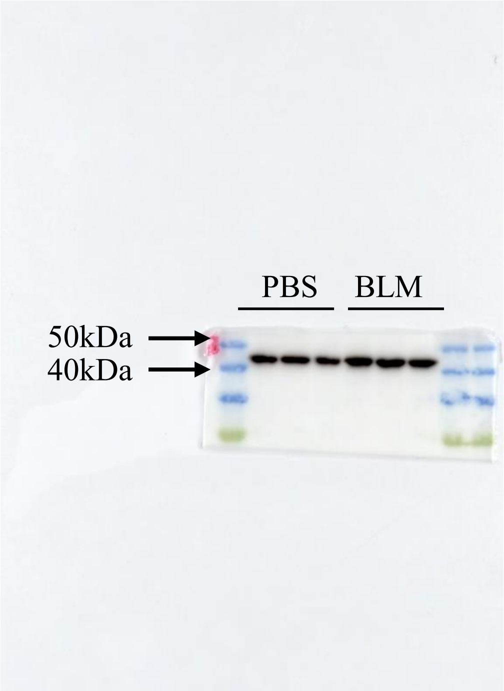

αSMA

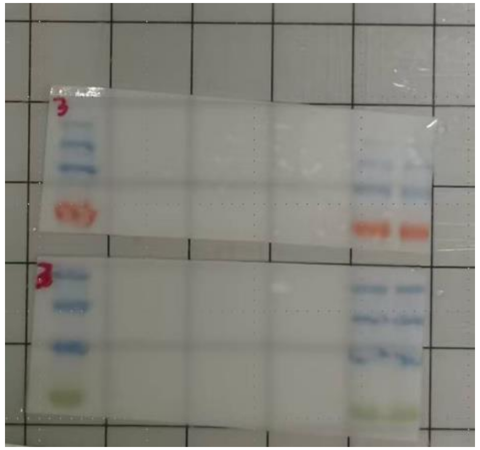

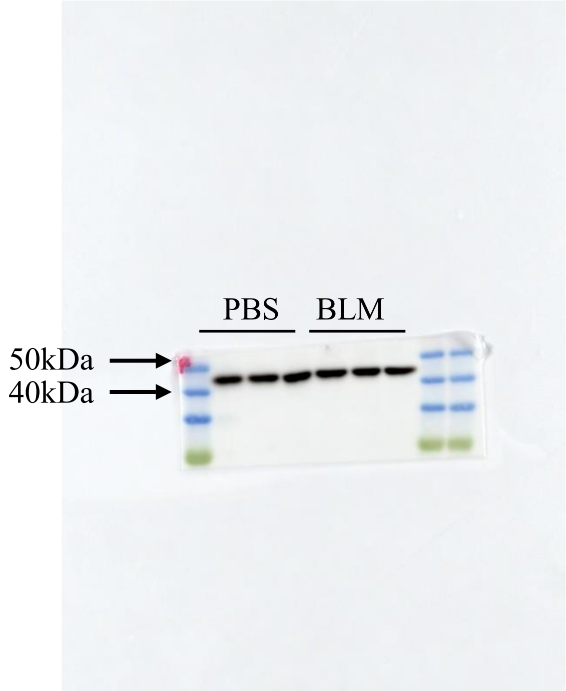

β-actin

Figure.S2I

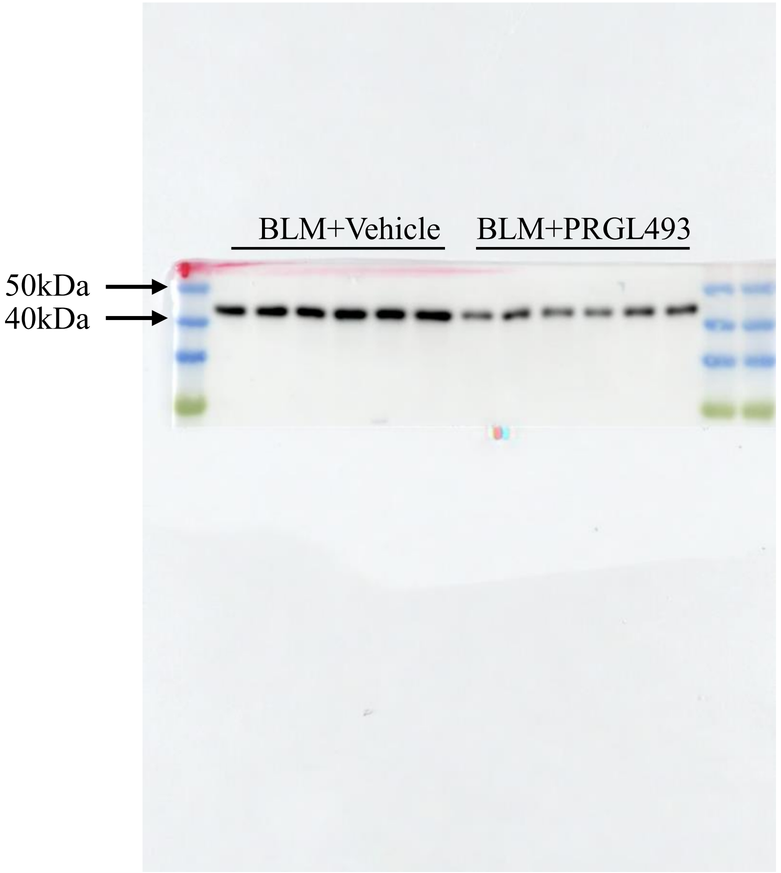

αSMA

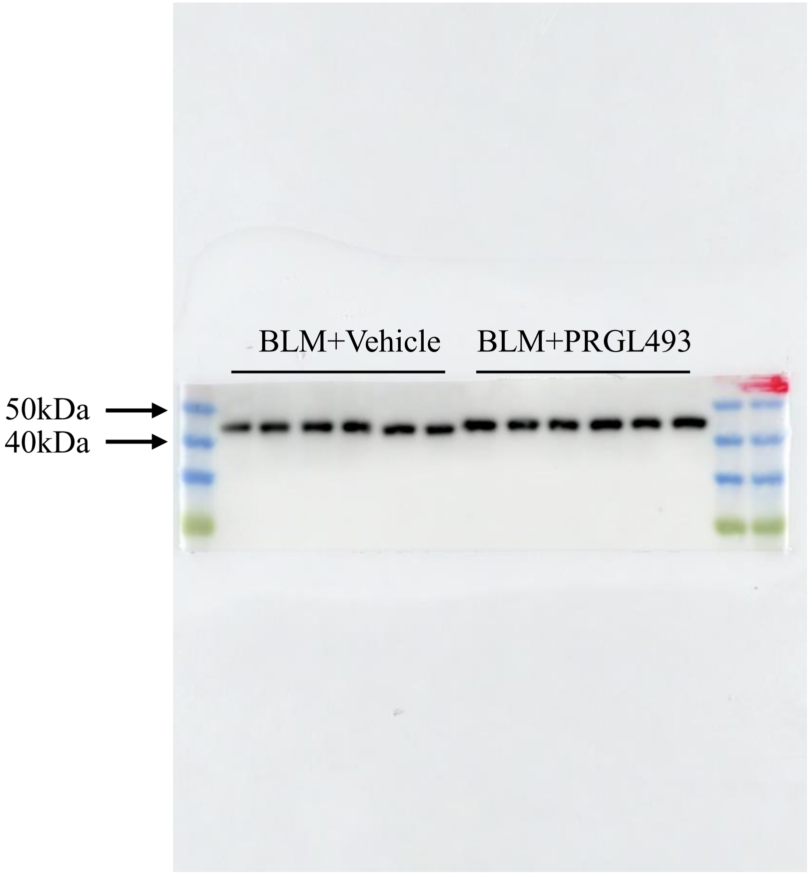

β-actin

Figure.S3I

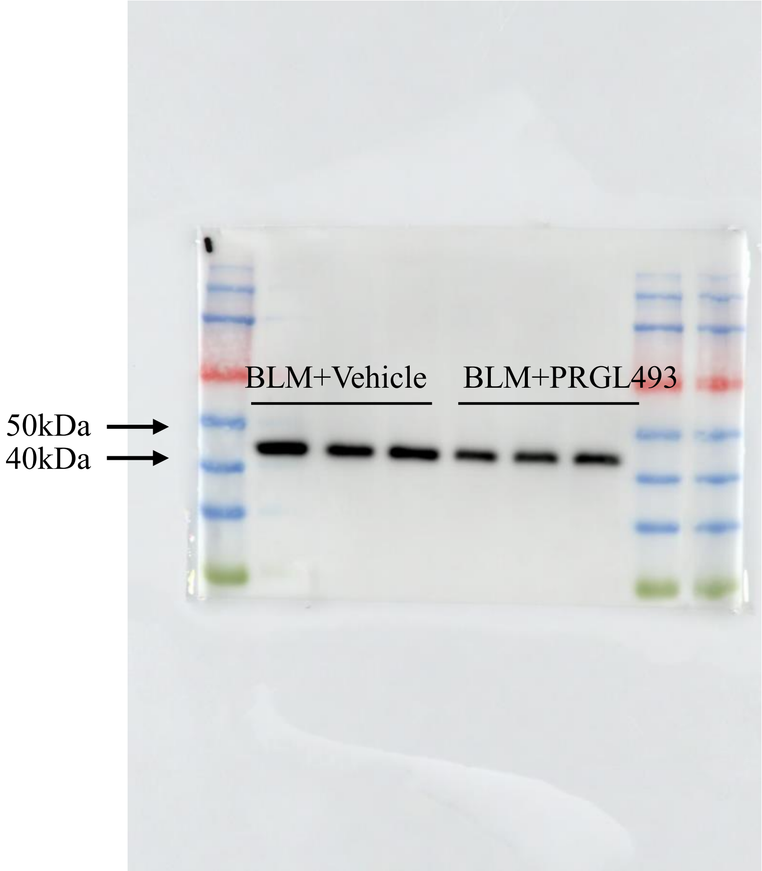

αSMA

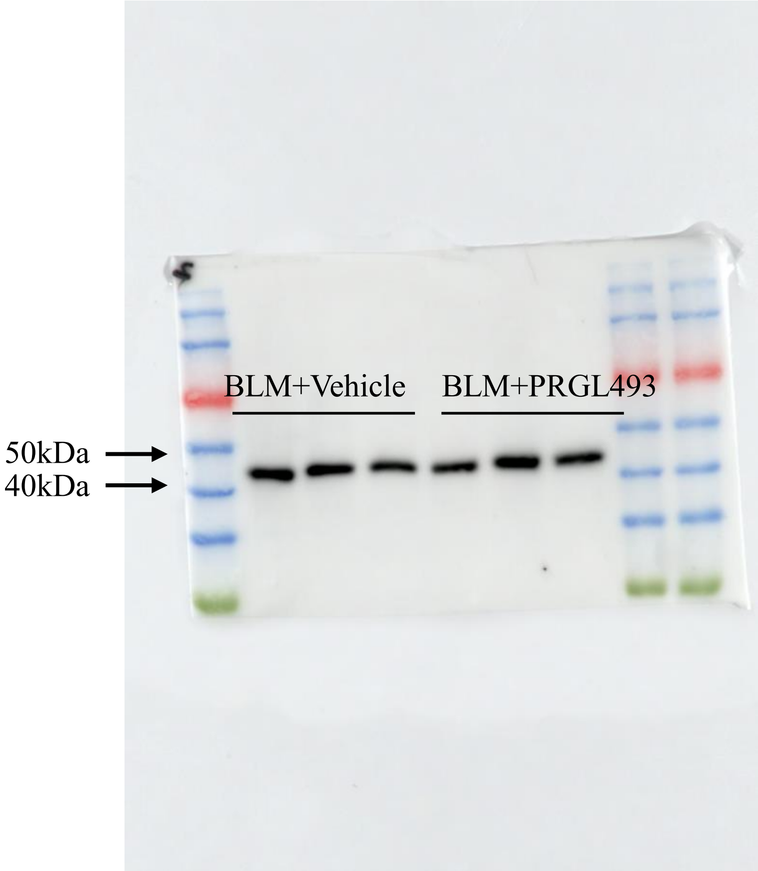

β-actin

Figure.S4B

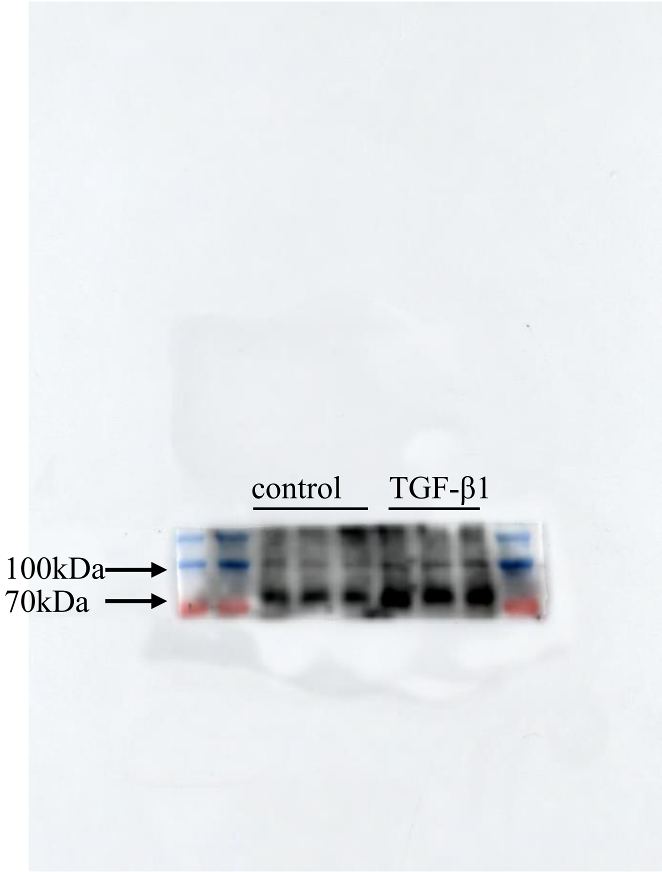

ACSL4

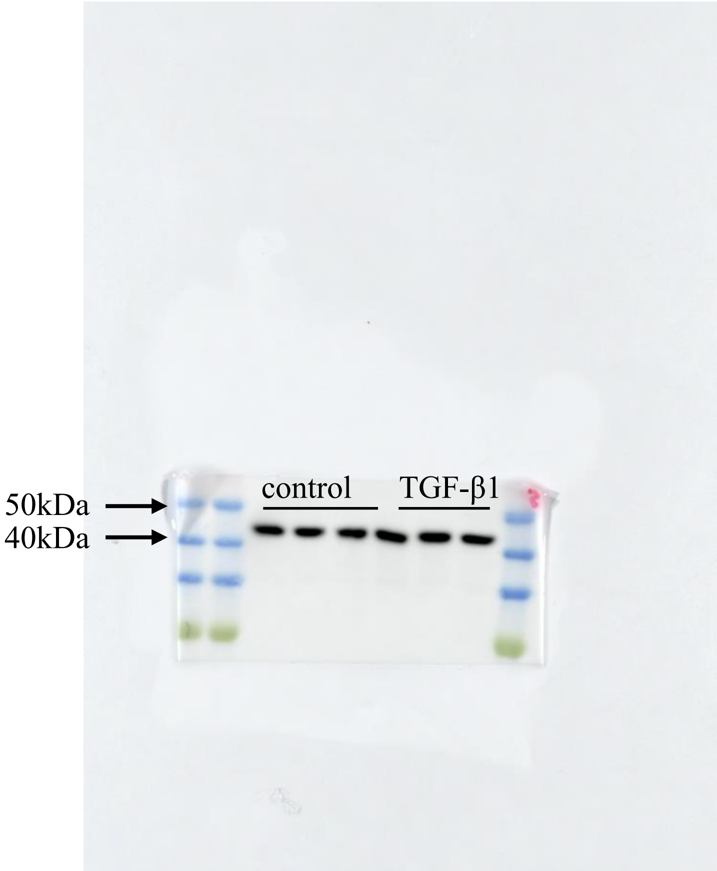

β-actin

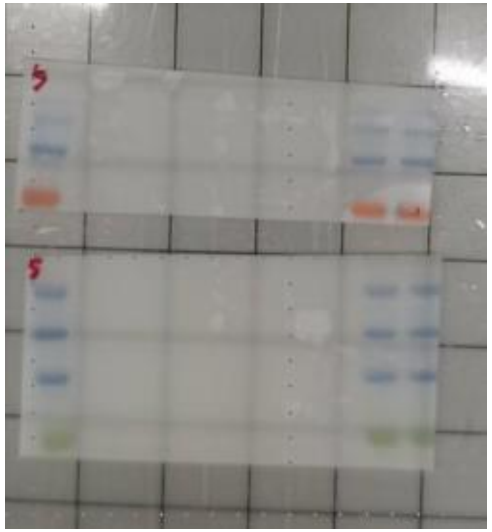

Figure.S4E

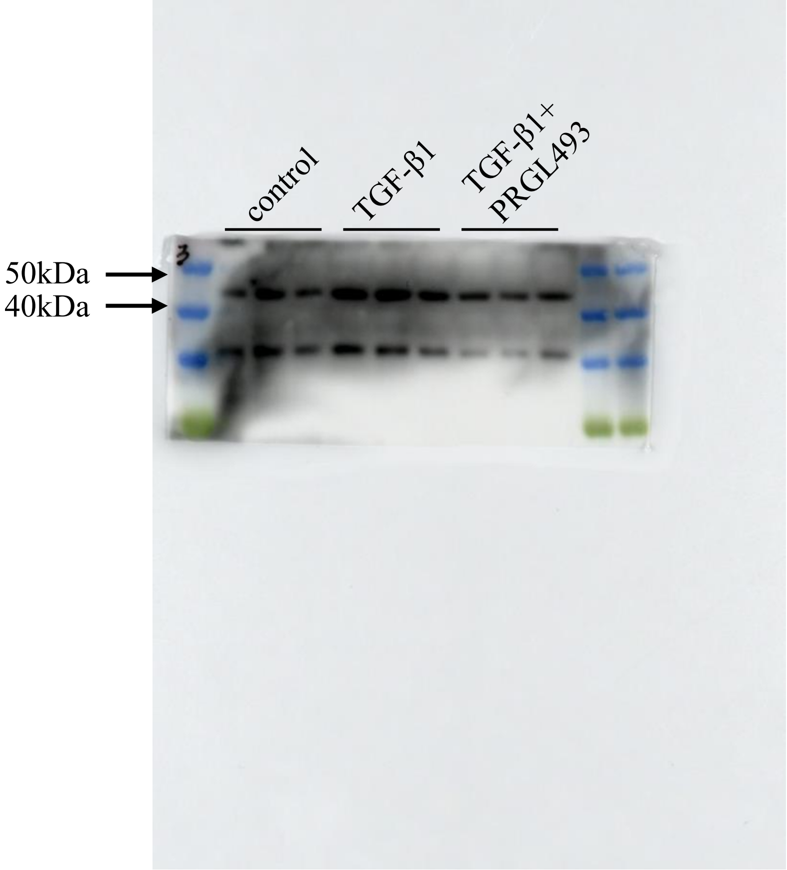

αSMA

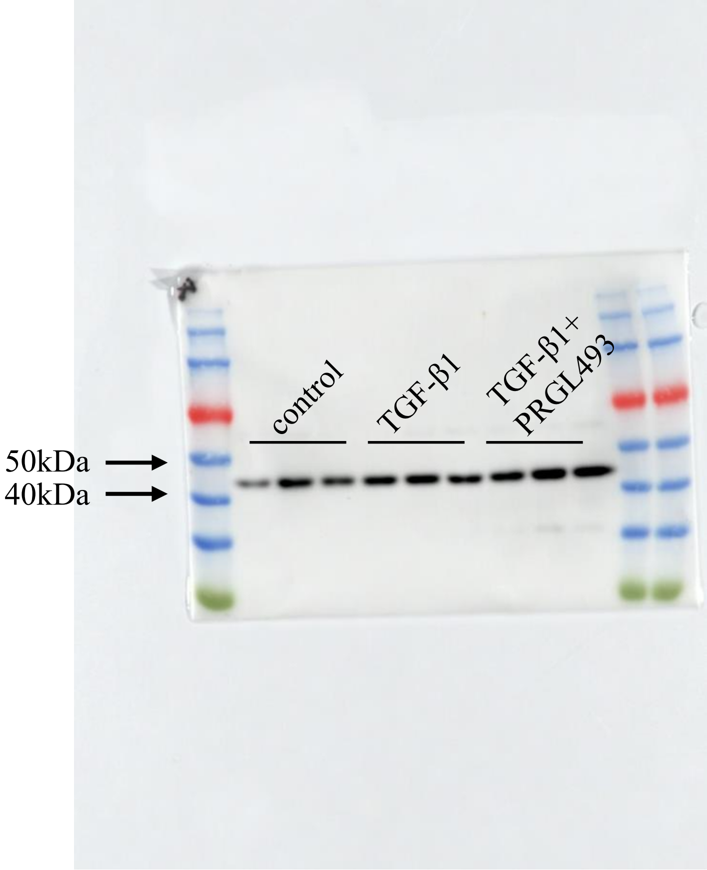

β-actin

Figure.S5N

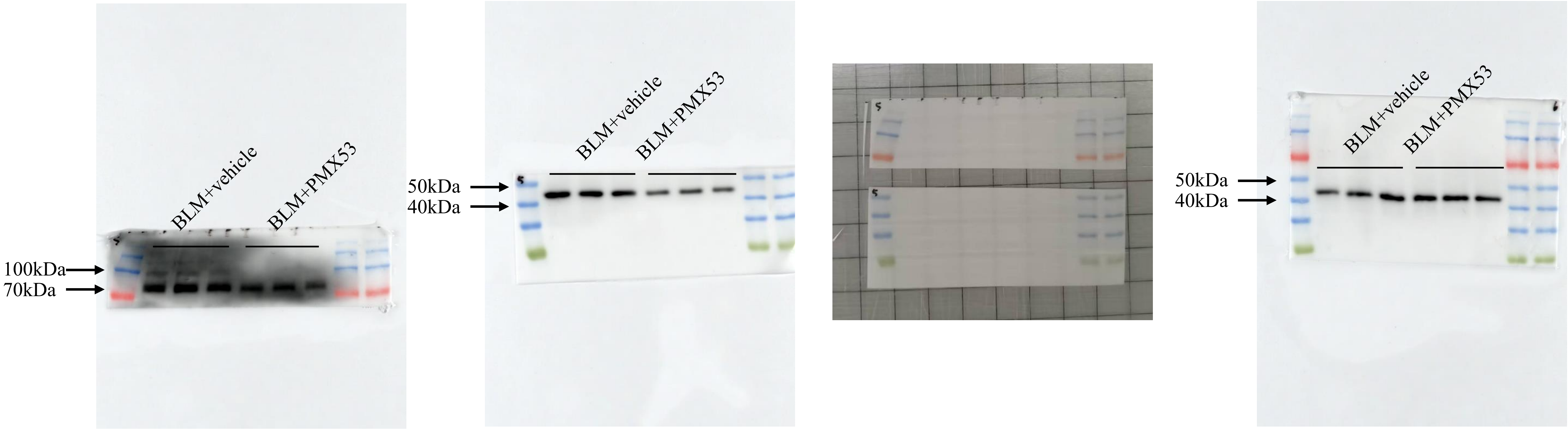

ACSL4 αSMA β-actin

Figure.S6N

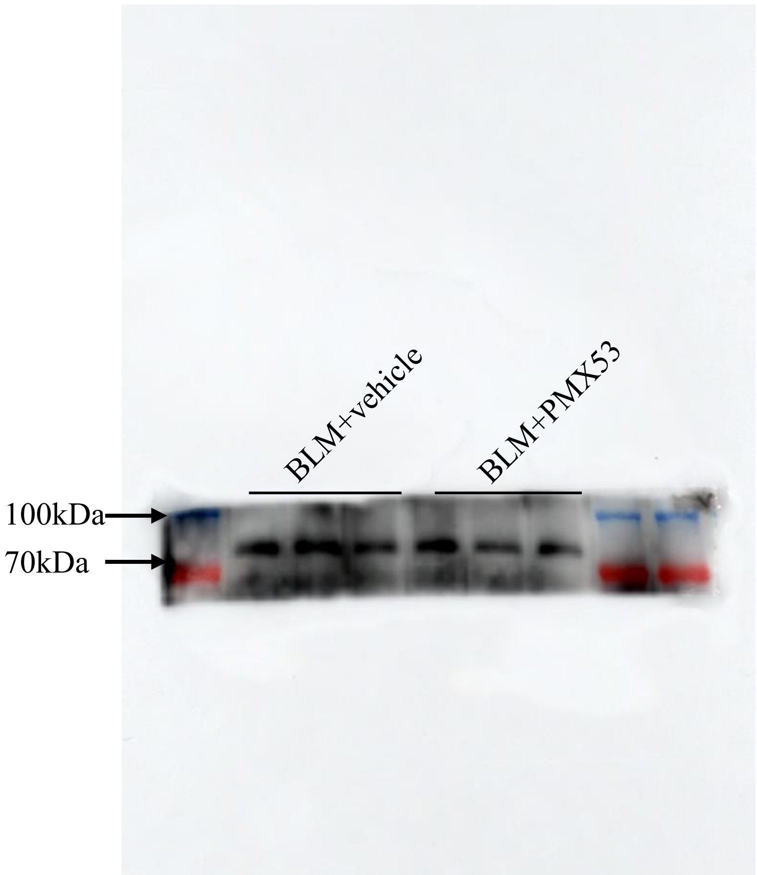

ACSL4

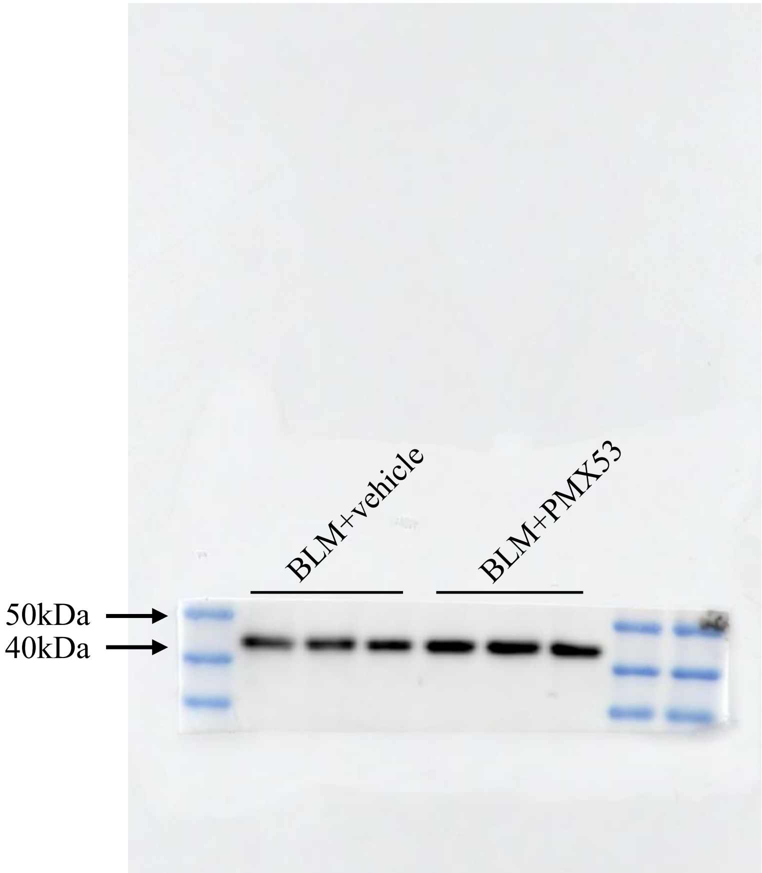

β-actin

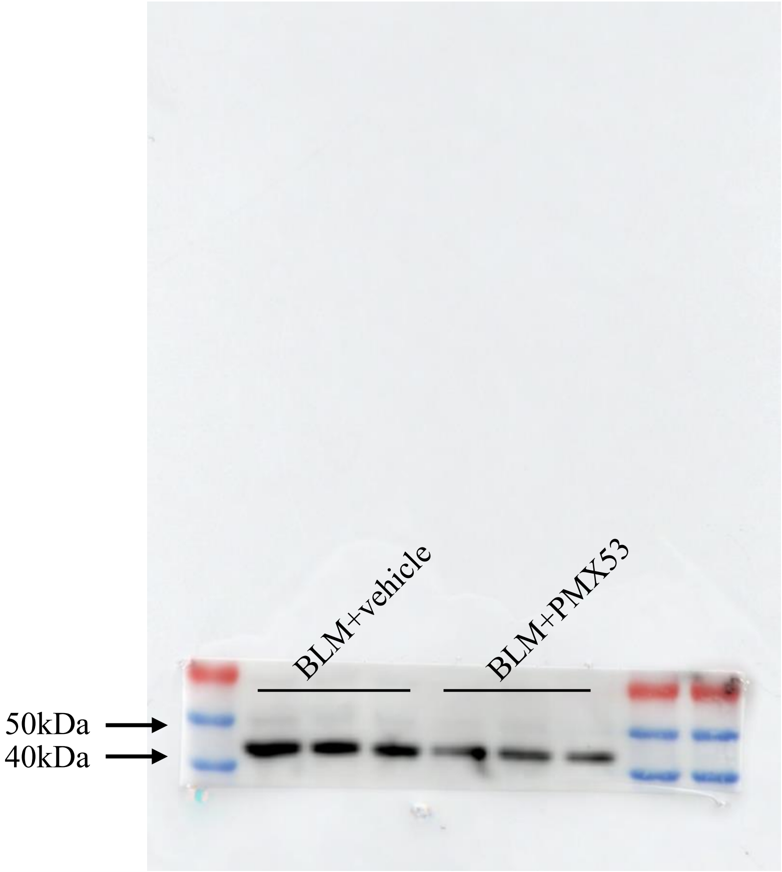

αSMA

Figure.S7B

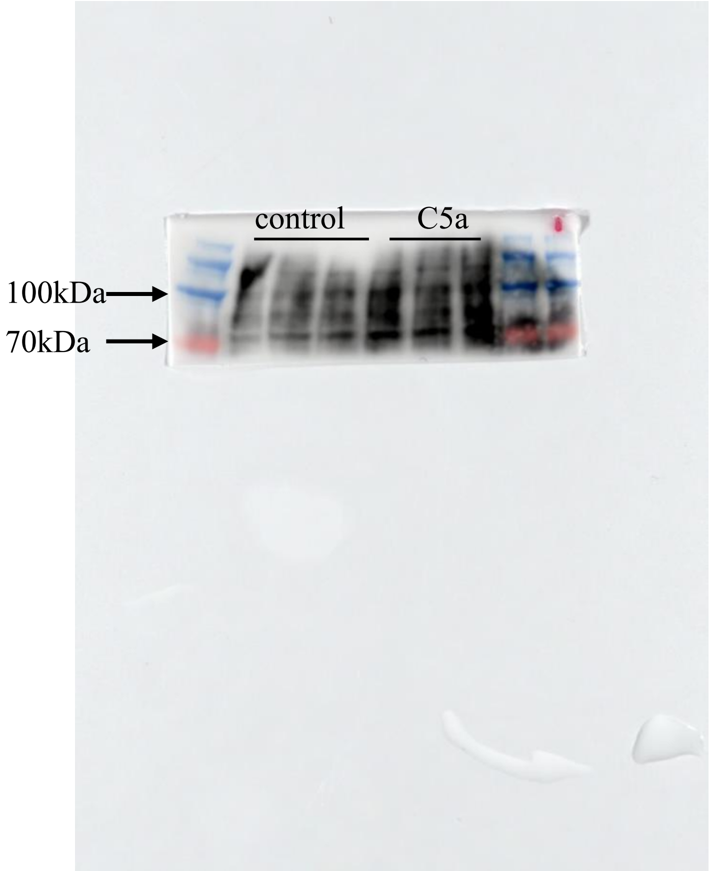

ACSL4

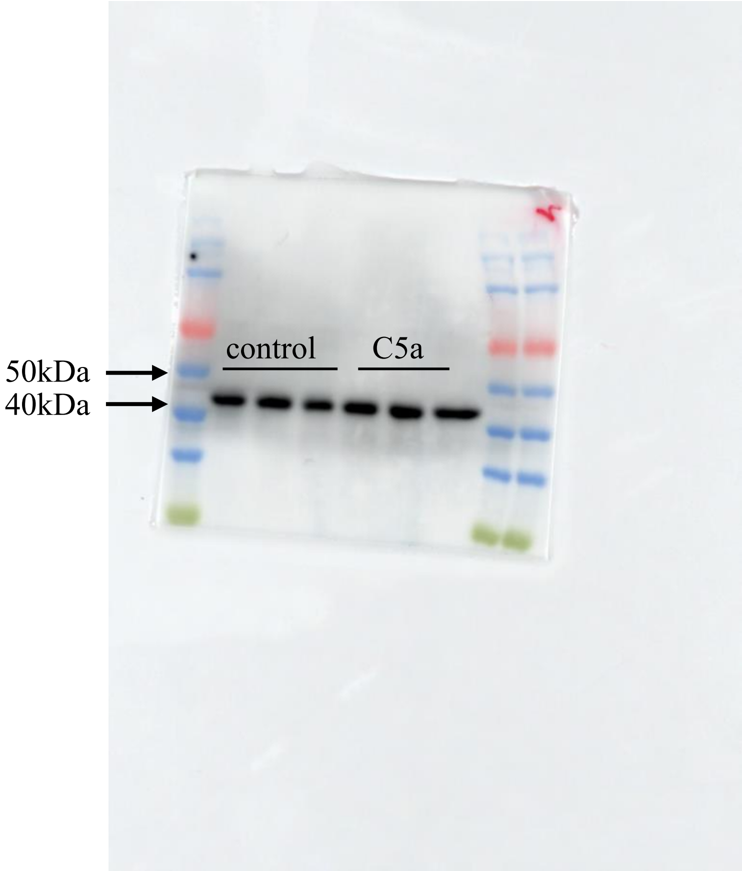

β-actin

Figure.S7E

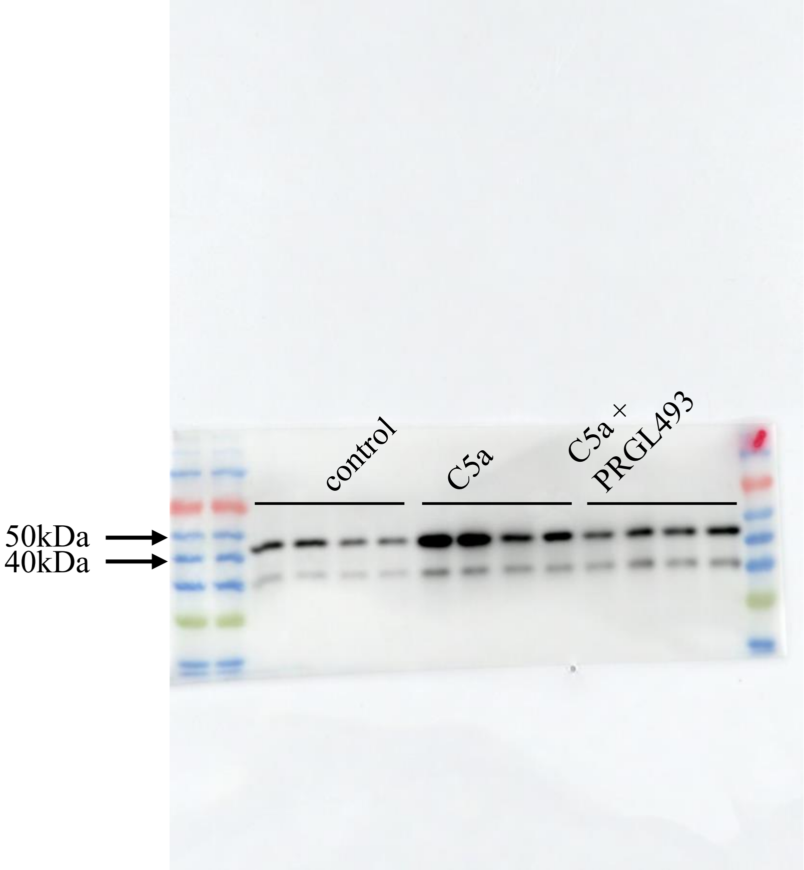

αSMA

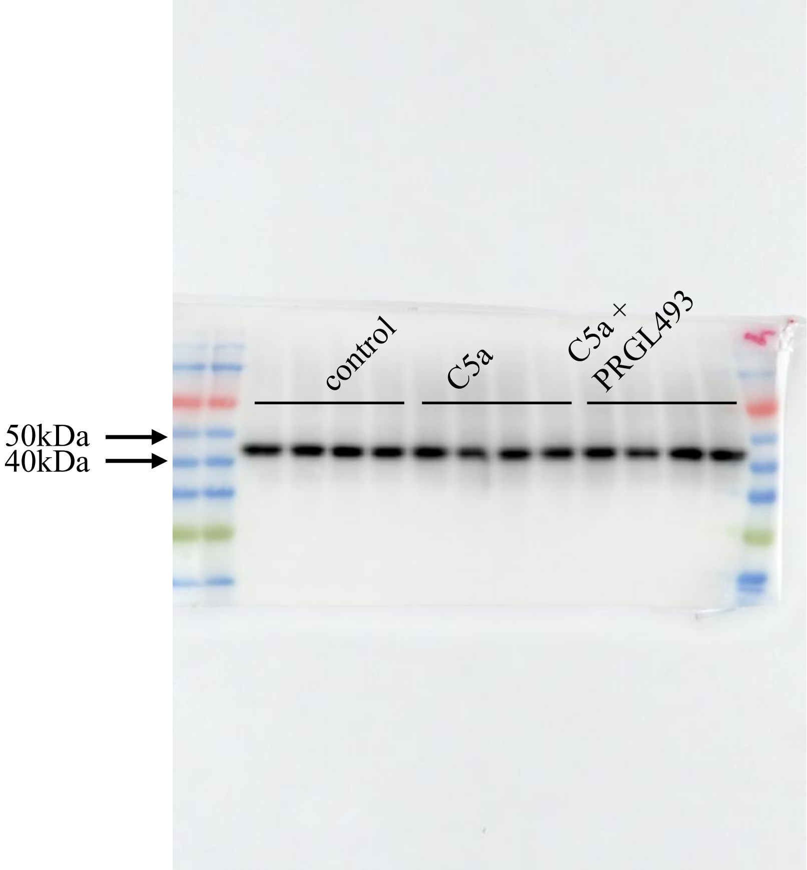

β-actin

Figure.S8D

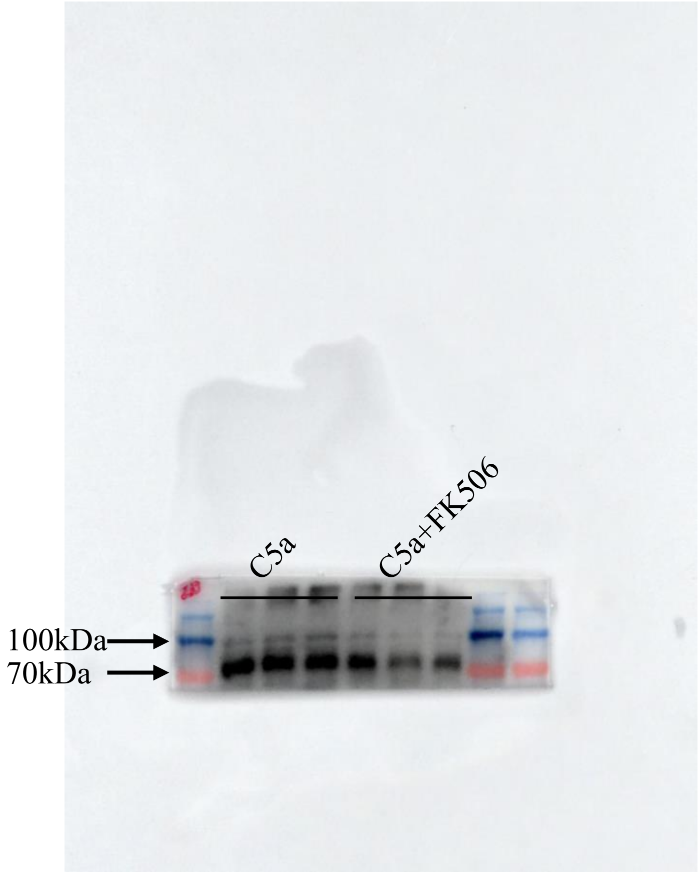

ACSL4

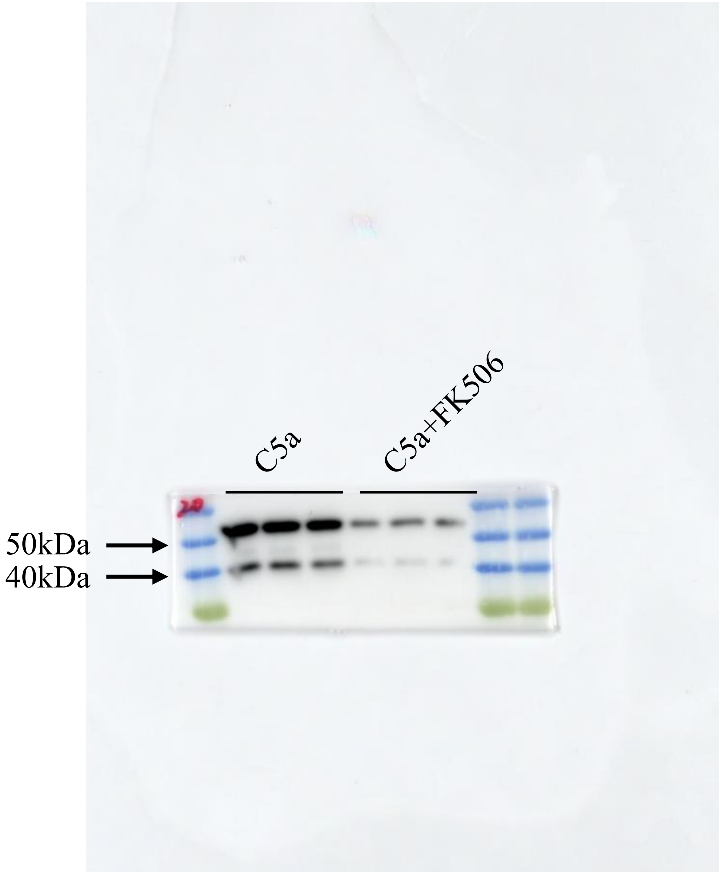

αSMA

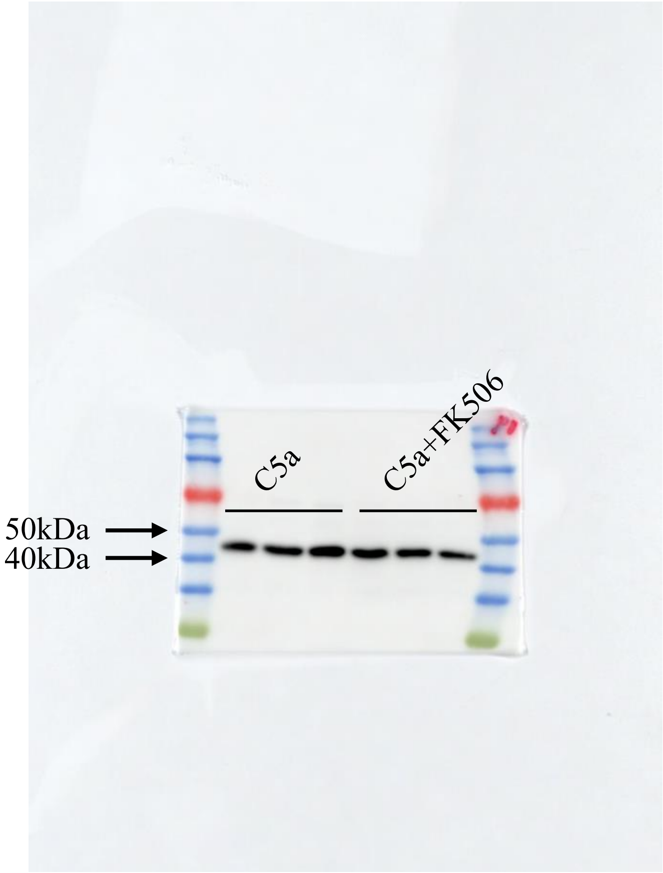

β-actin
