# Supplementary material for: ACSL4 Drives C5a/C5aR1–Calcium-Induced Fibroblast-to-Myofibroblast Transition in a Bleomycin-Induced Mouse Model of Pulmonary Fibrosis
Source: Biomolecules. 2025 Jul 31;15(8):1106. doi: 10.3390/biom15081106 (PMC12383839; doi:10.3390/biom15081106)
Supplement: Supplementary file 1 [file biomolecules-15-01106-s001.zip › biomolecules-3689094-supplementary-v3-done.pdf]

biomolecules

MDPI

ACSL4 drives C5a/C5aR1-calcium-induced fibro-blast-to-myofibroblast transition in a bleomycin-induced mouse model of pulmonary fibrosis

Tingting Ren, Jia Shi, Lili Zhuang, Ruiting Su, Yimei Lai and Niansheng Yang

Supplementary figure legend and Supplementary table

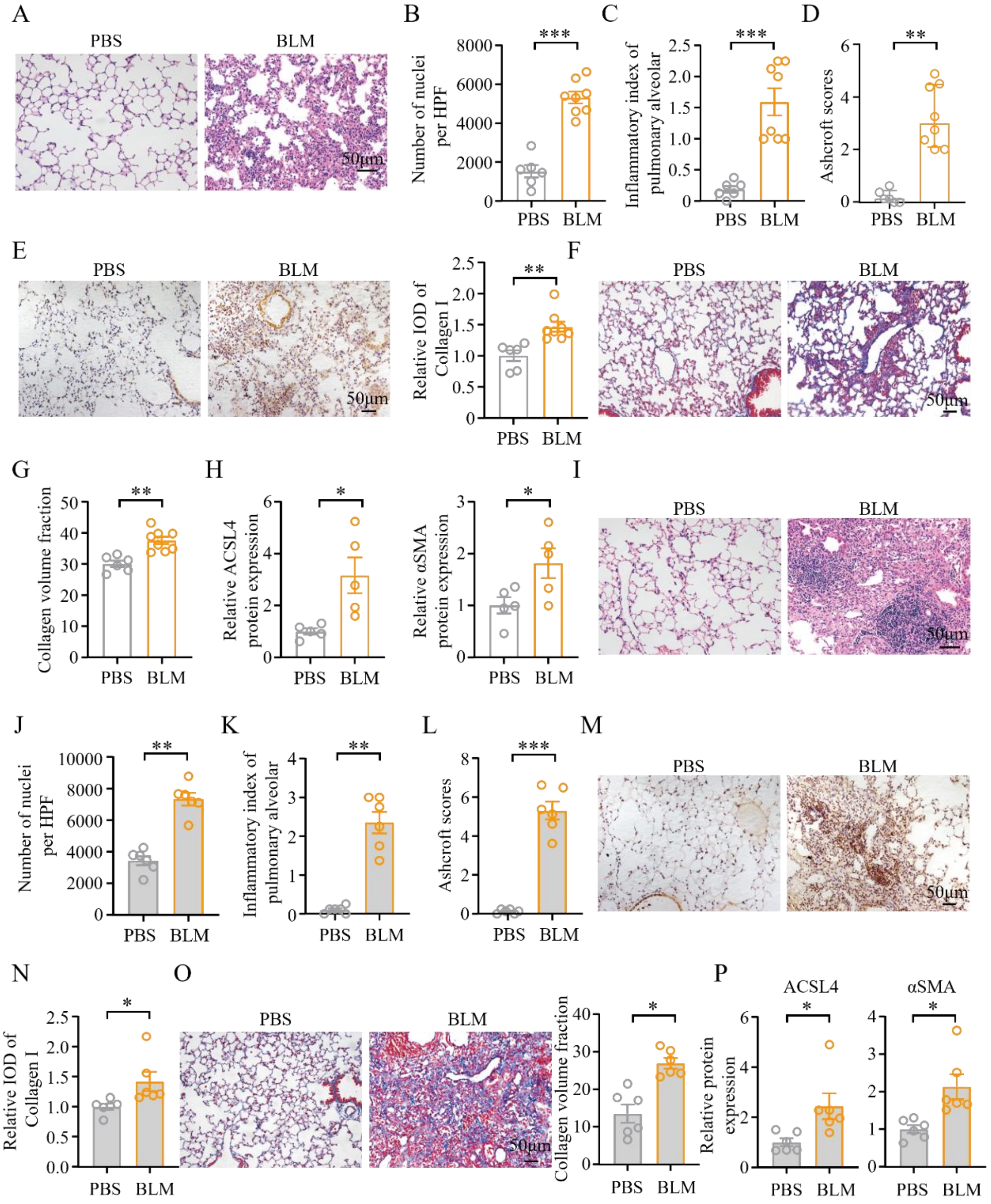

Figure S1. Pathological changes of lung tissue at early and chronic stage of pulmonary fibrosis. (**A**) HE staining of lung tissue of the mice. Representative images are shown. Original magnification: 200×. (**B**) Number of nuclei per high-power field of lung tissue of the mice. n=6-8 for each group. (**C**) Alveolar inflammatory score of the mice. n=6-8 for each group. (**D**)The Ashcroft score of the mice. n=6-8 for each group. (**E**) Immunohistochemical staining of collagen I in lung tissue of the mice. Representative images and relative IOD analyses are shown. Original magnification: 200×. n=6-8 for each group. (**F-G**) Masson's trichrome staining of lung tissue of the mice. Representative images and collagen volume fraction analyses are shown. Original magnification:200×. n=6-8 for each group. (**H**) The immunoblot analysis of ACSL4 and αSMA protein in lung tissues of the mice. Statistical plots are shown. n=5 for each group. (**I**) HE staining of lung tissue of the mice. Representative images are shown. Original magnification: 200×. (**J**) Number of nuclei per high-power field of lung tissue of the mice. n=6 for each group. (**K**) Alveolar inflammatory score of the mice. n=6 for each group. (**L**) Ashcroft score of the mice. n=6 for each group. (**M-N**) Immunohistochemical staining of collagen I of lung tissue of the mice. Representative images and relative IOD analyses are shown. Original magnification: 200×. n=6 for each group. (**O**) Masson's trichrome staining of lung tissue of the mice. Representative images and collagen volume fraction analyses are shown. Original magnification:200×. n=6 for each group. (**P**) The immunoblot analysis of ACSL4 and αSMA protein in lung tissues of the mice. Statistical plots are shown. n=6 for each group. All data are mean ± SEM. *p<0.05, **p<0.01, ***p<0.001 by Student's t-test.

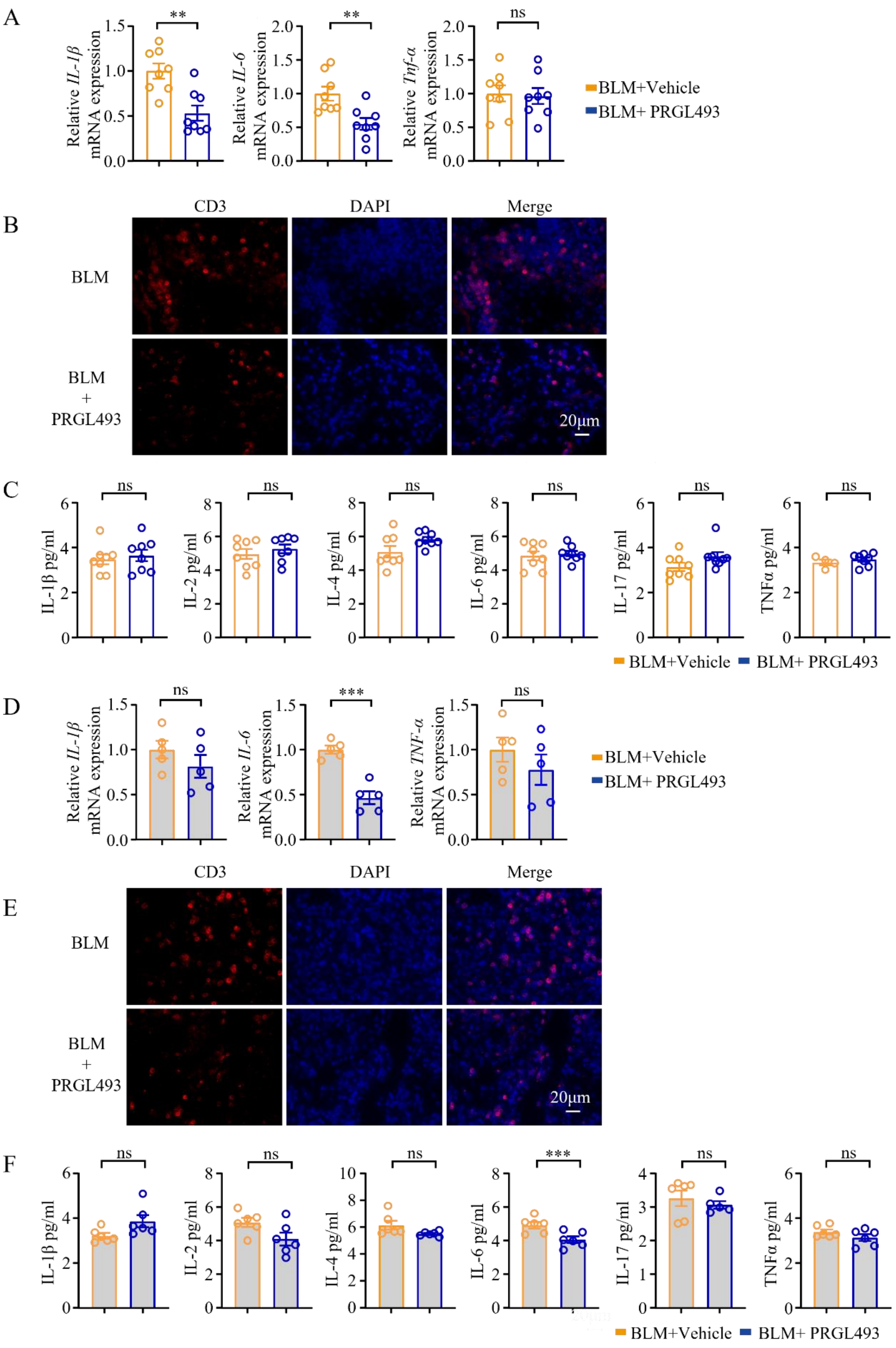

Figure S2. Expression of inflammatory factors in lung tissue and peripheral blood on day 7 and day 21 after bleomycin modeling. (**A**) Gene expression of *Il-1β*, *Il-6* and *Tnf-α* in lung tissue of the mice was quantified by qPCR. n=8 for each group. (**B**) Representative images of immunofluorescence staining of lung sections. CD3 (red), DAPI (blue). (**C**) Cytokine expression of IL-1β, IL2, IL-4, IL-6, IL-17, and TNF*α* in peripheral blood was quantified by Magnetic bead particle luminescence. n=4-8 for each group. (**D**) Gene expression of *Il-1β*, *Il-6*, and *Tnf-α* in lung tissues was quantified by qPCR. n=5 for each group. (**E**) Representative images of immunofluorescence staining of lung sections. CD3 (red), DAPI (blue). (**F**) Cytokine expression of IL-1β, IL2, IL-4, IL-6, IL-17, and TNF*α* in

peripheral blood was quantified by Magnetic bead particle luminescence. n=5-6 for each group. All data are mean ± SEM. **p<0.01, ***p<0.001 by Student's t-test. ns= no significant.

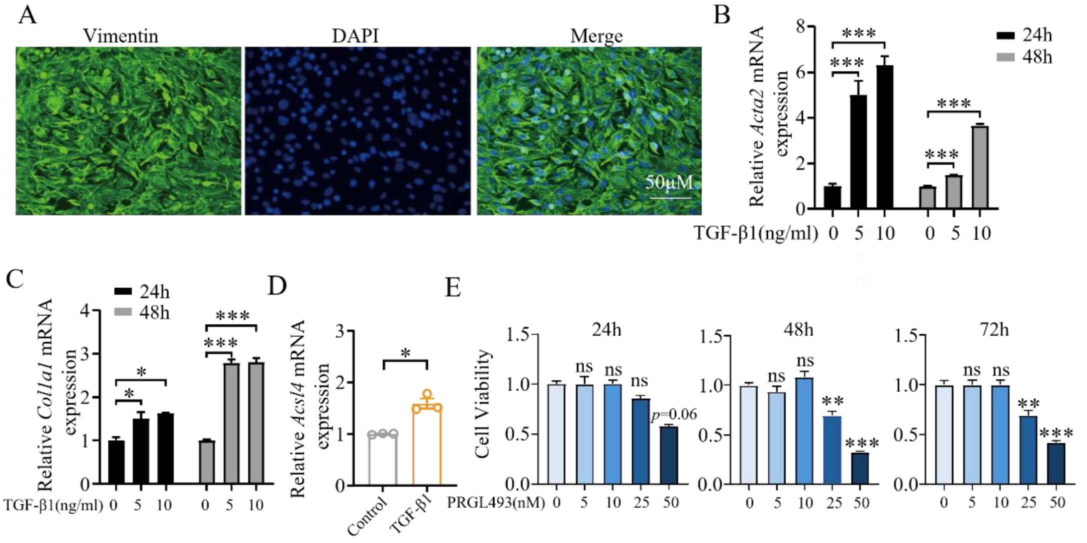

Figure S3. Identification of lung fibroblast, measurement of the concentration of TGF-β1 and PRGL493. (**A**) The expression of Vimentin in extracted cells. Representative immunofluorescence images are shown, Vimentin (green), and DAPI (blue). Original magnification:100×. (**B-C**) Gene expression of *Acta2* and *Col1a1* in lung fibroblasts was tested by qPCR after being treated with different concentrations of TGF-β1. n=4 for each group. (**D**) Gene expression of *Acsl4* in lung fibroblast was quantified by qPCR after being treated with TGF-β1 for 48h. n=3 for each group. (**E**) The effect of PRGL493 on the viability of lung fibroblasts was tested by CCK8 assay. n = 4 for each group. All data are mean ± SEM. *p<0.05, **p<0.01, ***p<0.001 by Student's t-test in panel D, or one-way ANOVA followed by adjustments for multiple comparisons in panels B, C and E. ns= no significant.

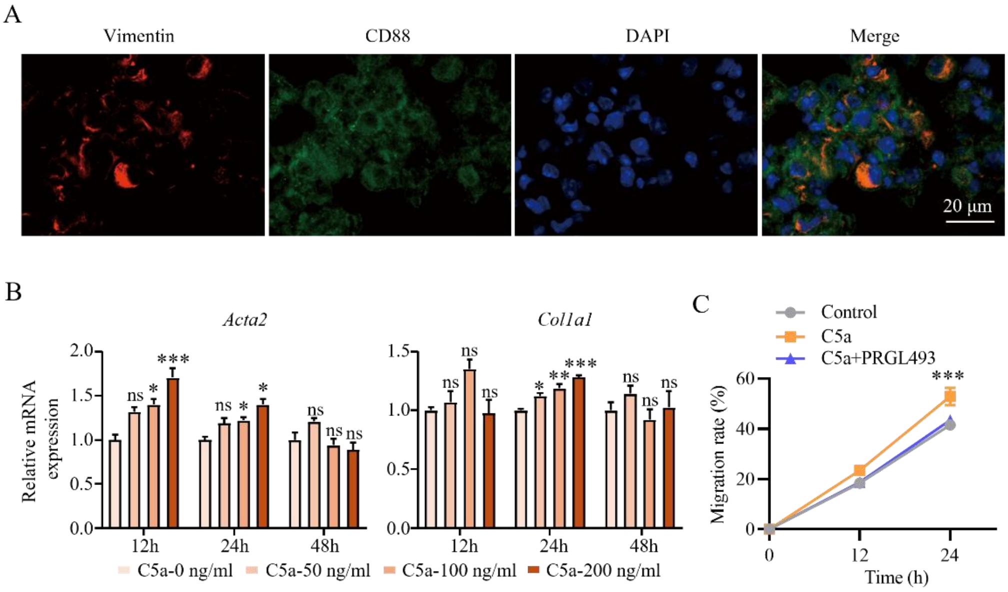

Figure S4. The expression of CD88 in lung fibroblasts and induction of lung fibroblasts by C5a. (**A**) The expression of Vimentin and CD88 in extracted cells. Representative immunofluorescence images are shown, Vimentin (green), CD88 (red), and DAPI (blue). Original magnification:400×. (**B**) Gene expression of *Acta2* and *Col1a1* in lung fibroblasts was tested by qPCR after being treated with different concentrations of C5a. n=4 for each group. (**C**) Lung fibroblast migration after different treatments was assessed by cell scratch assay. Statistical plots are shown. n=4 for each group. All data are mean ± SEM. *p<0.05, **p<0.01, ***p<0.001 by one-way ANOVA followed by adjustments for multiple comparisons in panel B, or two-way ANOVA followed by adjustments for multiple comparisons in panel C. ns= no significant.

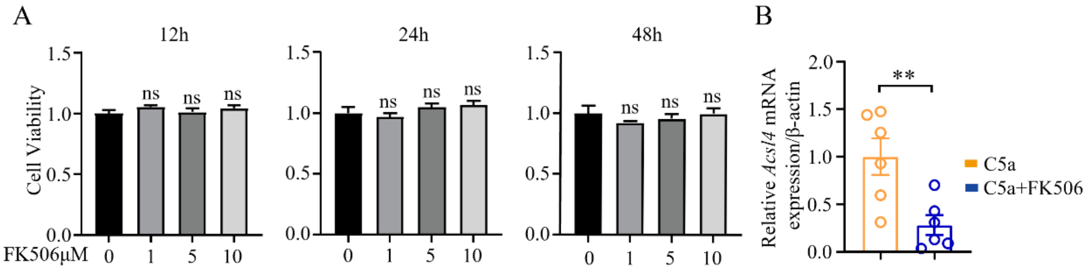

Figure S5. The Effect of FK506 on cell viability and the expression of ACSL4. (**A**) The effect of FK506 on the viability of lung fibroblasts was tested by CCK8 assay. n = 3-4 for each group. (**B**) Gene expression of *Acsl4* in lung fibroblasts was tested by qPCR. n=6 for each group. All data are mean ± SEM. **p<0.01 by one-way ANOVA followed by adjustments for multiple comparisons in panel A, or Student's t-test in panel B. ns = no significant.

Supplemental Table S1. Primer for qPCR. .

**Primer**		**Primer sequence（5'-3'）**
β-actin	F	GCAGGAGTACGATGAGTCCG
β-actin	R	ACGCAGCTCAGTAACAGTCC
ACSL4	F	ACTGGCGATATTGGAGAAT
ACSL4	R	CACATAGGACTGGTCACTT
ACTA2	F	ACACGGCATCATCACCAACT
ACTA2	R	ATTTTCTCCCGGTTGGCCTT
COL1A1	F	CCTGGCAAAGACGGACTCAA
COL1A1	R	GCTGAAGTCATAACCGCCAC
COL3	F	ATTCTGCCACCCCGAACTC
COL3	R	TGTCCACCAGTGCTTACGTG
FN1	F	AATCGTGCAGCCTCAATCCC
FN1	R	CAGGCTTGCTCTCGCAGTTA
TIMP1	F	CATCTCTGGCCTCTGGCATC-
TIMP1	R	CATAACGCTGGTATAAGGTGGTCTC
MMP2	F	CACCAAGAACTTCCGATTATCC
MMP2	R	TGCCATCAAAGACAATGTCCT
IL-1β	F	GGCTGCTTCCAAACCTTTGACC
IL-1β	R	CTTCTTCAAAGATGAAGGAAAAGAAGGTGC
IL-6	F	GAGACTTCCATCCAGTTGCCTTCT
IL-6	R	CTCTTTTCTCATTTCCACGATTTCCC
TNFα	F	TGGCCAACGGCATGGATCTCA
TNFα	R	CCCTCAGGGGTGTCCTTGGG
